# Enhancing endometrial receptivity: the roles of human chorionic gonadotropin in autophagy and apoptosis regulation in endometrial stromal cells

**DOI:** 10.1186/s12958-024-01205-x

**Published:** 2024-04-04

**Authors:** Bin Wang, Mingxia Gao, Ying Yao, Haofei Shen, Hongwei Li, Jingjing Sun, Liyan Wang, Xuehong Zhang

**Affiliations:** 1 The First School of Clinical Medicine, Lanzhou University, Lanzhou, China; 2 Reproductive Medicine Center, The First Hospital of Lanzhou University, Lanzhou, China; 3 Key Laboratory for Reproductive Medicine and Embryo, Gansu Province, Lanzhou, China; 4 Medical Laboratory Center, The First Hospital of Lanzhou University, Lanzhou, China

## Abstract

Inadequate endometrial receptivity often results in embryo implantation failure and miscarriage. Human chorionic gonadotropin (hCG) is a key signaling molecule secreted during early embryonic development, which regulates embryonic maternal interface signaling and promotes embryo implantation. This study aimed to examine the impact of hCG on endometrial receptivity and its underlying mechanisms. An exploratory study was designed, and endometrial samples were obtained from women diagnosed with simple tubal infertility or male factor infertile (*n* = 12) and recurrent implantation failure (RIF, *n* = 10). Using reverse transcription-quantitative PCR and western blotting, luteinizing hormone (LH)/hCG receptor (LHCGR) levels and autophagy were detected in the endometrial tissues. Subsequently, primary endometrial stromal cells (ESCs) were isolated from these control groups and treated with hCG to examine the presence of LHCGR and markers of endometrial receptivity (HOXA10, ITGB3, FOXO1, LIF, and L-selectin ligand) and autophagy-related factors (Beclin1, LC3, and P62). The findings revealed that the expressions of receptivity factors, LHCGR, and LC3 were reduced in the endometrial tissues of women with RIF compared with the control group, whereas the expression of P62 was elevated. The administration of hCG to ESCs specifically activated LHCGR, stimulating an increase in the endometrial production of HOXA10, ITGB3, FOXO1, LIF and L-selectin ligands. Furthermore, when ESCs were exposed to 0.1 IU/mL hCG for 72 h, the autophagy factors Beclin1 and LC3 increased within the cells and P62 decreased. Moreover, the apoptotic factor Bax increased and Bcl-2 declined. However, when small interfering RNA was used to knock down LHCGR, hCG was less capable of controlling endometrial receptivity and autophagy molecules in ESCs. In addition, hCG stimulation enhanced the phosphorylation of ERK1/2 and mTOR proteins. These results suggest that women with RIF exhibit lower levels of LHCGR and compromised autophagy function in their endometrial tissues. Thus, hCG/LHCGR could potentially improve endometrial receptivity by modulating autophagy and apoptosis.

**Supplementary Information:**

The online version contains supplementary material available at 10.1186/s12958-024-01205-x.

## Introduction

 Successful embryo implantation necessitates a well-prepared endometrial environment. The endometrium must undergo dynamic transformations at the cellular and molecular levels, transitioning from a “nonreceptive” to a “receptive” state. Poor endometrial receptivity is a critical factor leading to implantation failure, affecting approximately one-third of healthy women and 60-70% of infertile patients undergoing in vitro fertilization (IVF) treatment [1–3]. Signal transduction between the embryo and the endometrium is imperative during implantation. The key step in this transformation is the decidualization of the endometrium, especially the endometrial stromal cells (ESCs) [4], which play a pivotal role in mediating the receptivity of the endometrium for implantation [5]. Human chorionic gonadotropin (hCG), a key signaling molecule secreted by early embryos, plays an integral role in establishing and maintaining pregnancy [6–8]. hCG not only stimulates progesterone synthesis via its luteotropic effect but also regulates the function of the uterus and trophoblast cells during embryo implantation [9]. A study has reported that intrauterine hCG infusion can markedly enhance the live birth rate, ongoing pregnancy rate, clinical pregnancy rate, and implantation rate in women with infertility while decreasing the abortion rate [10]. This effect is linked to the role of HCG in promoting the expressions of endometrial receptivity markers, such as LIF, VEGF, IL-1/6/8, and TNF, thereby regulating estrogen synthesis and maintaining endometrial cellular immune balance [11–14]. In summary, hCG enhances endometrial receptivity via multiple pathways, providing a novel strategy for enhancing the rate of successful implantation.

hCG exerts its impact on the endometrium via the luteinizing hormone (LH)/hCG receptor (LHCGR) [15]. It is a member of the G protein-coupled receptor family and possesses an extracellular domain that binds to hCG or LH and an intracellular domain that facilitates signal transduction [16, 17]. This receptor is ubiquitously expressed in reproductive organs and related cells [18]. Upon binding to the ligand, LHCGR activates adenylate cyclase, in turn leading to elevated cAMP levels and gene activation [19]. Although previous studies have confirmed the presence of functional LHCGR in the human endometrium, the association between its endometrial expression and endometrial receptivity warrants further investigation.

ERK is a serine/threonine protein kinase and a member of the MAPK family [20]. Several studies have observed that ERK1/2 phosphorylation (p-ERK1/2) can regulate cell proliferation and activity, thereby influencing endometrial receptivity. This kinase This kinase plays a role in both endometrial epithelial cells (EECs) and stromal cells (ESCs), and promotes endometrial cell proliferation, differentiation, and vascular permeability [21, 22]. Furthermore, ERK appears to be associated with mTOR [23], a regulator of EEC protein and DNA synthesis [24]. In addition, mTOR plays a central role in embryo implantation [25, 26]. A study has previously documented that ERK1/2 and mTOR signaling are activated during the implantation window, inducing the upregulation of LIF, heparin-binding epidermal growth factor, and homeobox gene miRNA expression. The transition of the endometrium to a receptive state is thus facilitated [27]. Inhibition of mTOR activity has been reported to decrease the expression of receptivity-related factors in the endometrium [28].

Intriguingly, mTOR is an established regulator of autophagy, which is a crucial mechanism for the maintenance of cellular homeostasis and plays diverse roles in endometrial cell functions. An investigation has stated that endometrial autophagy levels fluctuate during embryo implantation [29]. During normal pregnancy, the level of autophagy in endometrial cells is higher in the early stages of implantation, especially on the 1st and 2nd day post-implantation. Upon the completion of embryo implantation, it decreases significantly [30, 31]. Autophagy increases during decidualization, and inhibition of endometrial autophagy has been shown to decrease endometrial apoptosis and decidualization [32]. Nevertheless, certain studies have reported that autophagy-related markers are downregulated following mouse embryo implantation [33]. Thus, the precise role of autophagy in implantation needs to be examined further.

In this study, the influence of hCG on the endometrium and the link between autophagy molecules and embryo implantation were investigated. The impact of hCG on uterine molecules and morphology during the implantation window was assessed, and the phosphorylation levels of ERK1/2 and mTOR in hCG-induced molecular changes were analyzed.

## Materials and methods

### Patient selection and collection of human endometrial tissues

Human endometrial tissue samples used in this study were obtained from the Reproductive Center of the First Hospital of Lanzhou University. Our control group (*n* = 12) was composed of women aged 25–35 with simple tubal infertility or male factor infertile. Patients with recurrent implantation failure (RIF, *n* = 10) included those ≤ 35 years of age, had undergone > 3 cycles of embryo transfer, and had experienced 4–6 high-grade cleavage-stage embryo transfers or ≥ 3 high-grade blastocyst transfers, all of which resulted in unsuccessful implantation. The additional inclusion criteria were as follows: (1) Imaging examination revealed normal ovarian function and endometrial morphology. (2) Hysteroscopy was routinely performed prior to ovulation induction, and the findings of endometrial pathological examination were normal. (3) Serum sex hormone levels were within the normal range. (4) The semen analysis results of their male partners were normal. All participants had a regular menstrual cycle (26–35 days), a normal body mass index (BMI) of 20–25 kg/m^2^, and did not have a history of endometriosis or pelvic inflammatory disease. Moreover, none of them had received hormone therapy within the last 3 months, and their ovarian reserves were normal. Patient details are summarized in Table 1. Endometrial samples were collected aseptically from the uterine fundus using a sterile sampler during the mid-secretory phase of the menstrual cycle (6–8 days after LH surge). Each sample was categorized into three parts. While two of them were stored in Falcon tubes kept on ice and immediately sent to the laboratory for processing, the third one was fixed in formalin and used for standard histologic examination.

**Table 1:** Clinical characteristics of women enrolled in the present study

Variables	Normal secretory phase (*n* = 12)	RIF secretory phase (*n* = 10)	*P*-value
Age (y)	33.17 ± 3.71	33.50 ± 3.06	> 0.05
BMI (kg/m^2^)	21.76 ± 2.20	21.62 ± 1.75	> 0.05
Cycle length	28.00 (28.00–30.00)	28.00 (27.25-30.00)	> 0.05
Infertility time (y)	2.50 (2.00-5.25)	5.50 (4.00–7.00)	> 0.05
Basal FSH	6.65 (5.57–7.40)	6.30 (5.22–7.89)	> 0.05
Basal LH	5.42 ± 2.37	5.44 ± 2.09	> 0.05
Basal E_2_	41.40 (33.58–75.03)	47.45 (37.95–51.58)	> 0.05
Basal P	0.26 (0.15–0.37)	0.72 (0.34–3.33)	> 0.05
Basal PRL	21.04 ± 10.53	29.23 ± 10.62	> 0.05
Failure times	0.00 (0.00–0.00)	3.50 (3.00–5.00)	< 0.05
Implantation	2.00 (1.00–2.00)	7.00 (6.00–9.00)	< 0.05

Data are mean ± SD

*RIF* Recurrent implantation failure

### Isolation of primary endometrial stromal cells and cell culture

Endometrial tissues from the normal group were initially rinsed twice with phosphate-buffered saline (PBS). Subsequently, these tissues were minced into small pieces and digested in 0.5% type I collagenase (Coolaber, China) at 37 °C for 1 h. The suspension was then filtered through a 40-µm nylon sieve (Biosharp, China). Stromal cells, which passed through the sieve, were thoroughly rinsed with PBS and resuspended in Dulbecco’s Modified Eagle Medium (DMEM)/Nutrient Mixture F-12 supplemented with 10% fetal bovine serum and 1% penicillin-streptomycin agent. These primary ESCs were grown independently in Petri dishes and were collected only when the purity of the third-generation ESCs exceeded 95%. At this stage, the cells were grown until they achieved 70-90% confluence. Subsequently, they were serum-starved for 24 h before hCG treatment. Each cell culture experiment was replicated at least three times using different individual samples of ESCs.

### Cell counting kit-8 (CCK-8) assay

Cell viability was evaluated using the Cell Counting Kit-8 (CCK-8) assay (Abmole, USA) according to the manufacturer’s instructions. After drug treatment, 10µL of the CCK-8 solution was added to each well of the culture plate. The plate was then incubated in a culture incubator for 2 h. After incubation, the absorbance was measured at 450 nm using a microplate reader. This absorbance is directly proportional to the number of live cells, providing a quantitative measure of cell viability.

### Live cell imaging

After the ESCs in the cell culture bottle were fully grown, they were digested with trypsin and blown into a single-cell suspension, followed by inoculation in a 96-well plate at the density of 3 × 10^3^ cells/well. The cells were cultivated in a constant-temperature incubator at 37 °C and 5% CO_2_. After the cells adhered to the wall, different concentrations of hCG were added to the cell culture medium of each group. Subsequently, the operating program was followed, and Cytology 5 was used to perform dynamic cell counting detection, image acquisition, and analysis.

### Real-time qPCR

Total RNA was extracted using an RNA extraction kit (Tiangen, China). RNA (1ug) was reverse transcribed with a cDNA synthesis kit (Takara, Japan). Real-time quantitative PCR (RT-qPCR) was performed using TB Green Premix Ex Taq (Takara, Japan) and an AB Applied Biosystems machine (ABI, USA). Each reaction was performed with a total volume of 20 mL, which comprised 2×TB Green Premix Ex Taq (10µL), 5′ and 3′ primers (0.8µL), ROX reference dye (0.4µL), cDNA (2µL), and ddH_2_O (6µL). The reactions were performed in triplicate using GAPDH as the reference transcript and the following primers:

**Table:** 

	Forward	Reverse
GAPDH	GGAGCGAGATCCCTCCAAAAT	GGCTGTTGTCATACTTCTCATGG
LHCGR	GAGGCAATAAAGGAGCTCACC	GATGATCTCTTCTTTTGCTTCACAT
HOXA10	GGTTTGTTCTGACTTTTTGTTTCT	TGACACTTAGGACAATATCTATCTCTA
ITGB3	ACTTCTCCTGTGTCCGCTACAAG	GGTGTCAGTACGCGTGGTACA
LIF	TGCCAATGCCCTCTTTATTC	GTTGACAGCCCAGCTTCTTC
P62	CATCGGAGGATCCGAGTGTG	TTCTTTTCCCTCCGTGCTCC
LC3 II	AGCAGCATCCAACCAAAATC	CTGTGTCCGTTCACCAACAG

### Immunohistochemistry (IHC)

Endometrial biopsies were fixed in 4% paraformaldehyde, paraffin-embedded, and sliced. For deparaffinization, the sections were first heated at 60 °C for 30 min and then passed through a series of decreasing concentrations of alcohol. The slides were subsequently boiled in the preferred pH 6 for 10 min. To block endogenous peroxidase activity, the sections were incubated for 15 min with 1% hydrogen peroxide solution in methanol. They were then incubated for 30 min with 3% bovine serum albumin (BSA) to block nonspecific antibody binding and overnight at 4 °C with rabbit anti-LHCGR antibody (1:200 dilution; Proteintech) or rabbit anti-LC3 antibody (1:300 dilution; Proteintech). The sections were washed in PBS on the following day and incubated with anti-rabbit secondary antibody for 30 min. After DAB staining for 3 min, the nucleus was restained with hematoxylin for 1 min. The images were acquired using OLYMPUS BX51 and the ImageView software, and the relative OD was determined using the ImageJ software.

### Western blot analysis

Protein extract was performed on stromal cell cultures and tissue samples obtained from fertile women in the proliferative and secretory phase, and subsequently lysed and isolated. Protein concentrations were then measured using a BCA Protein Assay Kit (Boster, China). Equal amounts of denatured protein were separated via electrophoresis in 10% SDS polyacrylamide gels, then transferred to polyvinyl difluoride membranes (Millipore, Billerica, USA). These membranes were saturated with blocking buffer for 1 h. Following this, membranes were incubated with rabbit polyclonal antiLHCGR (1:1,000 dilution; Proteintech), rabbit polyclonal anti-HOXA10 (1:1,000 dilution; Proteintech), rabbit polyclonal anti-ITGB3 antibodies (1:2,000 dilution; Proteintech), rabbit polyclonal anti-LIF antibodies (1:1,000 dilution; Proteintech) and mouse monoclonal anti-MECA-79 antibodies (1:500 dilution; Santa Cruz) at 4 °C. The blots were then incubated with HRP-conjugated anti-rabbit IgG for 2 h. Finally, proteins were detected by the enhanced chemiluminescence (ECL) detection kit (Bio-Rad, USA) and visualized using the film exposure. Then analyzing gels and western blots with ImageJ.

### LHCGR knock down with siRNA

ESCs grown in 60-mm dishes or 6-well culture plates to 30–50% confluency were transfected with 100 nmol/dish of a nontargeting negative control siRNA (siRNA-NC) or LHCGR siRNA (Shanghai GenePharma Co., Ltd, China). The siRNA sequence was: siRNA-LHCGR sense: 5’-CUCUCUCACAAGUCUAUAUTT-3, antisense: 5’-AUAUAGACUUGUGAGAGAGT-T-3; siRNA-NC sense:5’-UUCUCCGAACGUGUCACGUTT-3, antisense: 5’-ACGUGAC-ACGUUCGGAGAATT-3. For this, GP-transfect-mate reagent was used (GenePharma, Shanghai, China), according to the manufacturer’s instructions. After treatment for 6 h with the siRNA, the medium containing the transfection reagents was removed and replaced with fresh medium for the following 24–72 h.

### Immunofluorescence (IF)

After in vitro treatment for the stipulated duration, the cells were fixed in paraformaldehyde and treated with 0.2% Triton solution to facilitate intracellular labeling. Subsequently, they were blocked with 5% BSA for 1-2 h at room temperature and incubated with the primary antibody against LHCGR (1:200 dilution; SAB), VIM (1:300 dilution; Bioss), CK18 (1:300 dilution; Bioss), or LC3 (1:400 dilution; Proteintech) at 4 °C. The cells were then incubated with the secondary antibody at room temperature for 60 min, washed, and treated with an antifade mounting medium containing DAPI (Solarbio, China).

### Apoptosis detection

Annexin V-FITC/PI apoptosis kit (MULTI SCIENCES, Hangzhou, China) was used to detect the apoptosis. During the process, ESCs were seeded into 6-well plates and incubated for 24 h then treated with 0.1IU/mL hCG for 72 h. Cells were harvested by trypsin and washed with 1×PBS twice. Then, 1 × 10^5^ cells were resuspended with 500µL binding buffer, and incubated with 5µL Annexin V-FITC and 10µL PI for 15 min at room temperature in the dark. Finally, we could immediately assess the apoptosis level by flow cytometry (BD, USA).

### Co-immunoprecipitation (Co-IP)

After the above treatment, ESCs were lysed with IP lysis buffer (25 mM Tris-HCl, 150 mM NaCl, 1 mM EDTA, 1% NP-40, and 1% protease inhibitor). The cell lysates were precleared with Protein A/G PLUS-Agarose (Thermo, USA) and then mixed with Bcl-2 (4ug, Proteintech) or control IgG (Proteintech, USA) for 1 h at 4 °C. The immunoprecipitates were captured on Protein A/G PLUS-Agarose and analyzed using a western blot with antibodies against Bcl-2 or Beclin1 (1:1000, Proteintech).

### Statistical analysis

The collected data was expressed as percentages or ratios relative to the corresponding negative controls. The values were presented as mean ± standard deviation (SD). Analysis of Variance (ANOVA) or an unpaired t-test was used to compare groups, as appropriate. The statistical analysis was performed using GraphPad Prism software (Version 8.1.1, California, USA). A *P*-value of less than 0.05 was considered statistically significant.

## Results

### Decreased expression of endometrial receptivity factors and LHCGR in patients with RIF

**Fig. 1:**
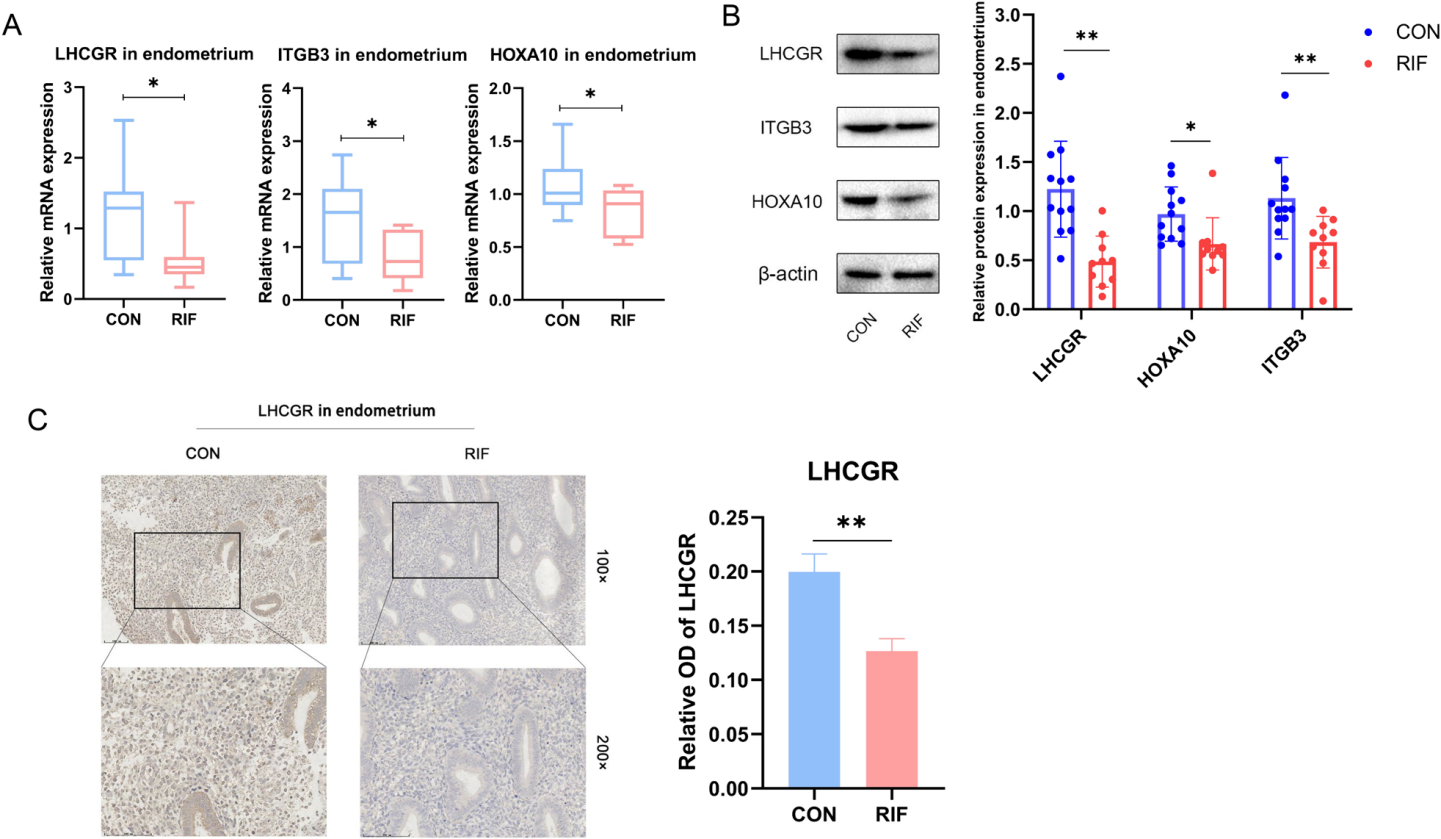
Endometrial receptivity is down-regulated in RIF patients. **A** The mRNA expression levels of HOXA10, ITGB3 and LHCGR in the endometrium of normal controls (*n* = 12) and RIF patients (*n* = 10). **B** WB was used to detect the protein levels of HOXA10, ITGB3 and LHCGR in endometrium of normal control (*n* = 12) and RIF patients (*n* = 10). Analyzing gels and western blots with ImageJ. Error bars, mean ± SD. *P < 0.05, **P < 0.01. **C** Representative IHC images of LHCGR localization in endometrial tissue from normal control (*n* = 12) and RIF patients (*n* = 10) in secretory phase. Scale, 100 μm and 50 μm, analyzing relative OD with ImageJ. Error bars, mean ± SD. **P < 0.01

The gene expression of receptivity-related molecules in various endometrial tissues was assessed using RT-qPCR. The findings signified that the mRNA expression levels of HOXA10, ITGB3, and LHCGR in the RIF group (*n* = 10) were significantly lower than those in the control group (*n* = 12) (*p* < 0.05) (Fig. 1A). These results were corroborated by western blotting, which showed that the protein levels of HOXA10, ITGB3, and LHCGR were consistently reduced in patients with RIF (*n* = 10) (Fig. 1B). IHC analysis confirmed the decreased expression of LHCGR in endometrial tissues and suggested its localization in stromal cells (Fig. 1C). These data highlight the potential role of LHCGR in establishing uterine receptivity.

### Downregulation of autophagy in the endometrium of patients with RIF

**Fig. 2:**
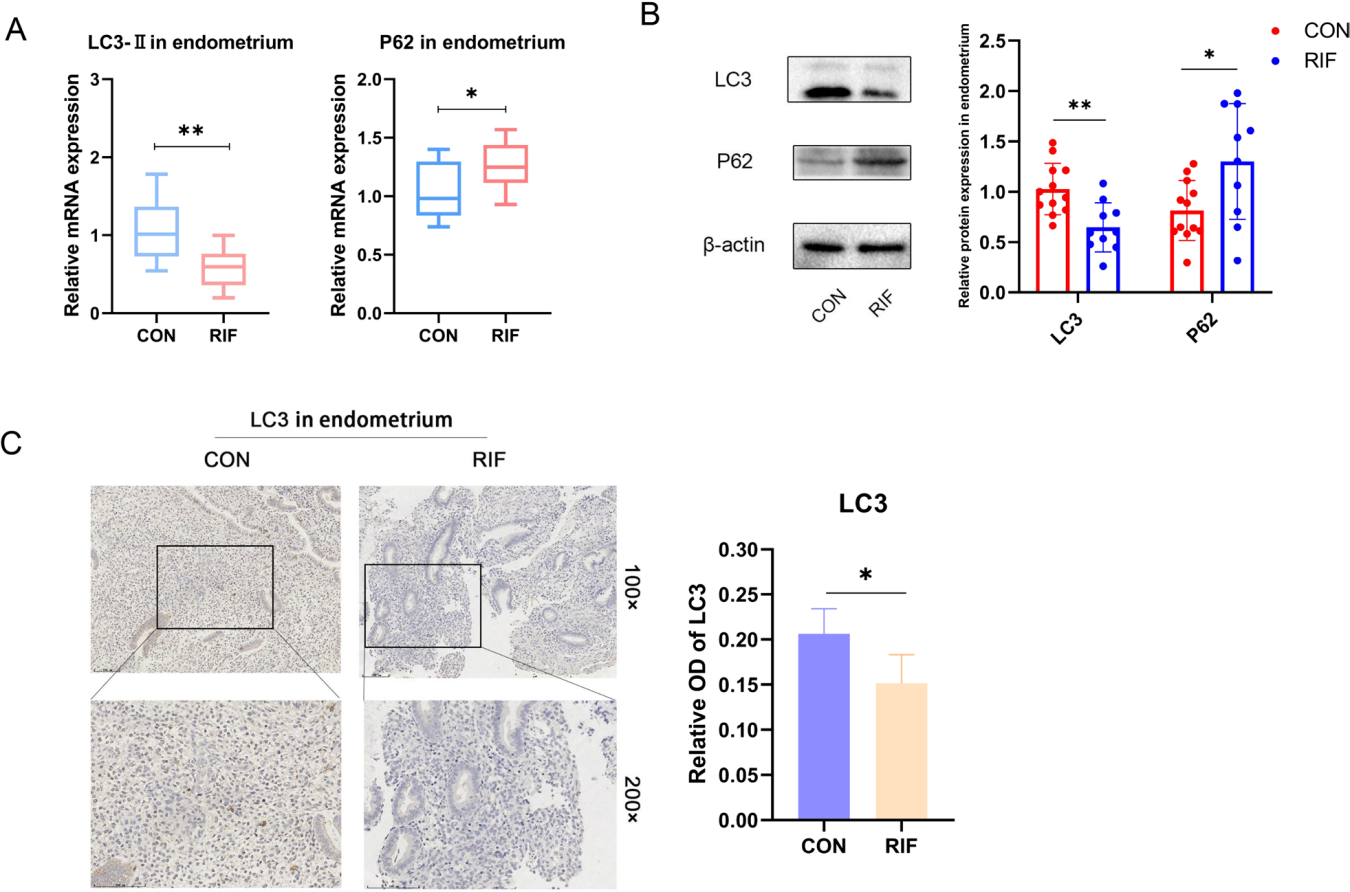
The level of endometrial autophagy in RIF patients was down-regulated. **A** mRNA expression levels of LC3-II and P62 in endometrium of normal control (*n* = 12) and RIF patients (*n* = 10). **B** Left: Western blotting was used to detect the protein levels of LC3B and P62 in the endometrium of normal controls (*n* = 12) and RIF patients (*n* = 10). Right: analyzing gels and western blots with Image J. Error bars, mean ± SD. *P < 0.05, **P < 0.01. **C** Representative IHC images of LC3 localization in endometrial tissue from normal control (*n* = 12) and RIF patients (*n* = 10) in secretory phase. Scale, 100 μm and 50 μm, analyzing relative OD with Image J. Error bars, mean ± SD. *P < 0.05, **P < 0.01

The expressions of autophagy-associated markers were investigated in different endometrial tissues. The findings indicated that LC3-II mRNA expression was decreased, whereas P62 expression was increased in the endometrium of patients with RIF (*n* = 10) (Fig. 2A). The protein levels of LC3 and P62 were assessed in patients with RIF using western blotting. In line with the gene-level findings, LC3 expression was decreased and P62 expression was increased (Fig. 2B), which implied impaired formation of autophagosomes. In IHC analysis, the expression of LC3 was found to be much lower in RIF samples during the secretory phase compared with women of childbearing age (Fig. 2C). These findings indicate a reduction in the level of autophagy in the endometrium of patients with RIF.

### hCG enhanced the viability of ESCs

**Fig. 3:**
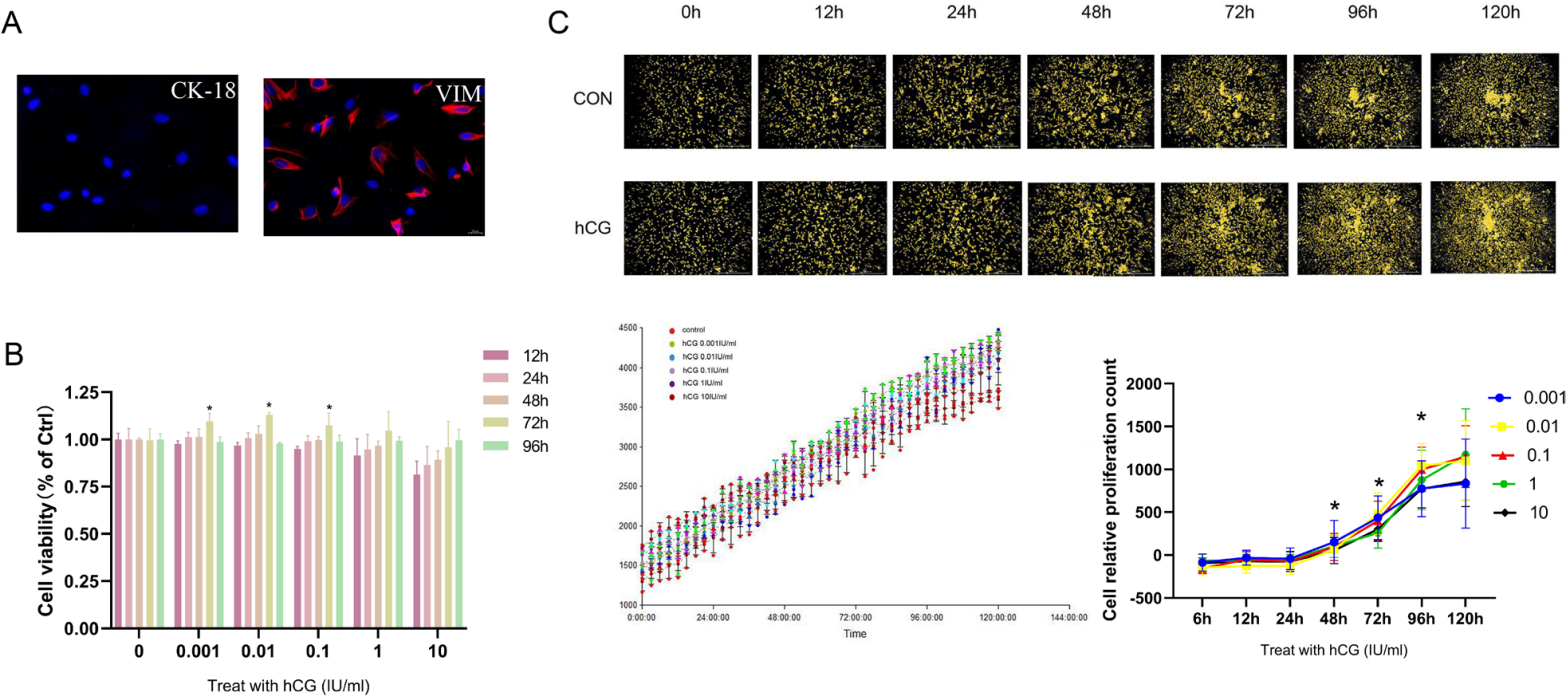
Identification and viability determination of primary ESCs. **A** IF showed the expression levels of VIM and CK-18 in the cytoplasm of primary ESCs, Scale, 20 μm. **B** hCG compared with the baseline and 0.001,0.01, 0.1 IU/ml hCG, *P* < 0.05. mean ± SD. **C** The effect of hCG on the proliferation of ESCs. The curve is the cell growth count of each group, and the line chart is the relative value-added growth curve of each group.*P < 0.05. mean ± SD. Scale: 1000 μm

To confirm cell purity, the expressions of cytokeratin-18 (CK-18) and vimentin (VIM) were evaluated in the cells. This approach aided in identifying primary ESCs. As CK-18 is predominantly found in EECs and VIM in ESCs, the latter can be identified by the differential expression of CK-18 and VIM. IF results demonstrated that VIM was present in the cytoplasm of the extracted cells (Fig. 3A) and CK-18 was almost absent, which asserted that the extracted and purified cells were primary ESCs. Subsequently, to assess the effect of hCG on endometrial viability, ESCs were exposed to different concentrations of hCG (10^−3^, 10^−2^, 10^−1^, 10^−0^, and 10^1^ IU/mL) for varying time intervals (12, 24, 48, 72, and 96 h). The viability of ESCs was determined using the CCK-8 assay. The findings suggested that as hCG concentration and exposure time increased, the viability of ESCs also increased (Fig. 3B). When hCG (10^−3^, 10^−2^, and 10^−1^ IU/mL) was administered for 72 h, the viability of ESCs reached its peak, and cell proliferation was significant (*p* < 0.05). Moreover, live cell imaging was performed to verify the effect of hCG treatment on ESCs. With the increase in time and concentration, hCG promoted the proliferation of stromal cells (Fig. 3C). Analysis of the cell count revealed that at 72 h, the administration of 10^−2^ and 10^−1^ IU/mL hCG significantly enhanced the proliferation rate of ESCs (*p* < 0.05). Therefore, in the follow-up study, the administration time of hCG was selected as 72 h. However, it should be noted that the highest concentrations of hCG were not conducive to the proliferation of ESCs, and the cells showed a trend of decreased viability.

### LHCGR expression increased after the hCG intervention

**Fig. 4:**
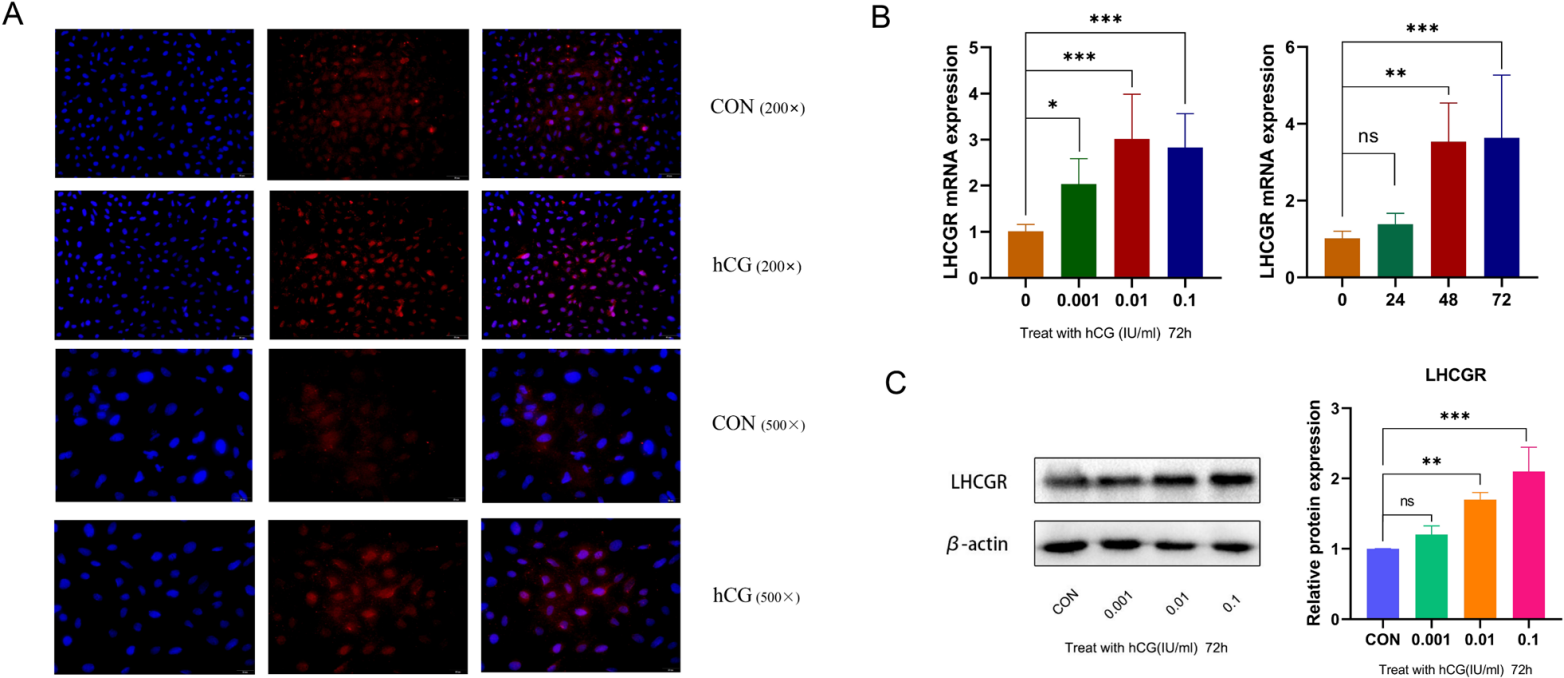
The expression level of LHCGR in ESCs. **A** The ESCs treated with hCG (0.1IU/mL, 72 h) was analyzed by immunofluorescence to determine the subcellular localization and protein expression level of LHCGR (red). The nuclei were stained with DAPI (blue). Scale, 50 μm and 20 μm. **B** The qRT-PCR analysis of LHCGR gene in ESCs after hCG treatment. *P < 0.05, **P < 0.01 (Student’s t-test). **C** Western blot analysis of LHCGR protein in ESCs after hCG treatment. *P < 0.05, **P < 0.01 (Student’s t-test)

The presence of LHCGR in ESCs was confirmed via IF. The specific markers were primarily located on the cell membrane and in perinuclear regions, and LHCGR expression was found to be elevated following hCG intervention (Fig. 4A). Subsequently, LHCGR expression was evaluated using RT-qPCR and western blot techniques. The results signified that hCG stimulation increased the expression of LHCGR in ESCs and that this change was concentration and time-dependent. Of these, the expression of LHCGR was significantly increased after 3 days of intervention with 0.1 IU/mL of hCG (*p* < 0.05, Fig. 4B and C). The varying expression of LHCGR with hCG treatment alludes that its expression in ESCs is specifically regulated by the concentration and time of hCG treatment.

### Endometrial receptivity molecules increased after hCG intervention

**Fig. 5:**
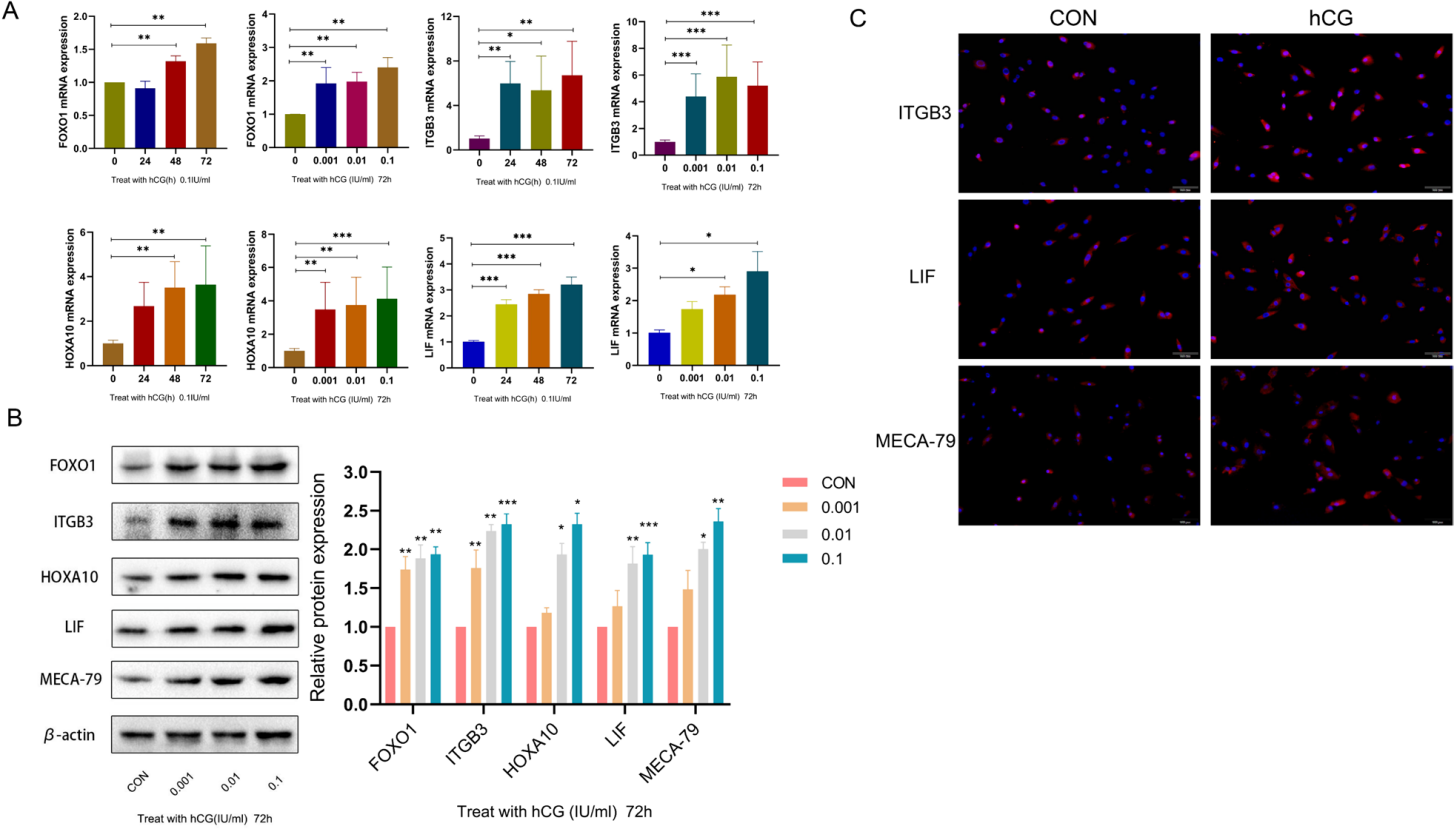
The expression levels of HOXA10, ITGB3, FOXO1, LIF and L-selectin ligand (MECA-79) in uterine ESCs after hCG intervention. **A** The mRNA expression levels of HOXA10, ITGB3, FOXO1 and LIF in ESCs were stimulated with hCG for 72 h. **B** The protein expression levels of HOXA10, ITGB3, FOXO1, LIF and L-selectin ligand (MECA-79) in ESCs were stimulated by hCG for 72 h. The data were shown as mean ± SD (*n* = 3): *P < 0.05, **P < 0.01, ***P < 0.001. **C** IF Representative images of ITGB3, LIF and L-selectin ligand (MECA-79) in ESCs treated with 0.1IU/mL hCG for 72 h. Scale: 100 μm

ESCs were deprived of nutrients overnight by culturing them in DMEM and were then stimulated with hCG at 37 °C. Subsequently, the effects on gene and protein expression levels of several molecules that have been reported to be regulated by hCG and/or associated with endometrial receptivity were assessed, including HOXA10, ITGB3, FOXO1, LIF, and L-selectin ligand (MECA-79). RT-qPCR results revealed that the expression levels of HOXA10, ITGB3, FOXO1, and LIF in ESCs were significantly increased after hCG administration compared with the control group (*p* < 0.05, Fig. 5A). Western blot analysis demonstrated that the protein expression levels of these molecules were also increased with the increase in hCG concentration. After treatment with 0.1 IU/mL hCG for 72 h, the expressions of tolerance factors differed significantly (*p* < 0.05, Fig. 5B). At the same time, we also used immunofluorescence to detect the expression and localization of ITGB3, LIF and L-selectin ligand. These molecules were previously confirmed to be expressed in the cytoplasm of endometrial epithelial cells. The results showed that in ESCs, ITGB3, LIF and L-selectin ligands were mainly expressed in the cytoplasm, and a small amount was expressed on the cell membrane. And after stimulation with 0.1 IU/mL hCG for 72 h, the immune reaction was enhanced (Fig. 5C).

### The Knockdown of LHCGR affected the function of hCG in ESCs

**Fig. 6:**
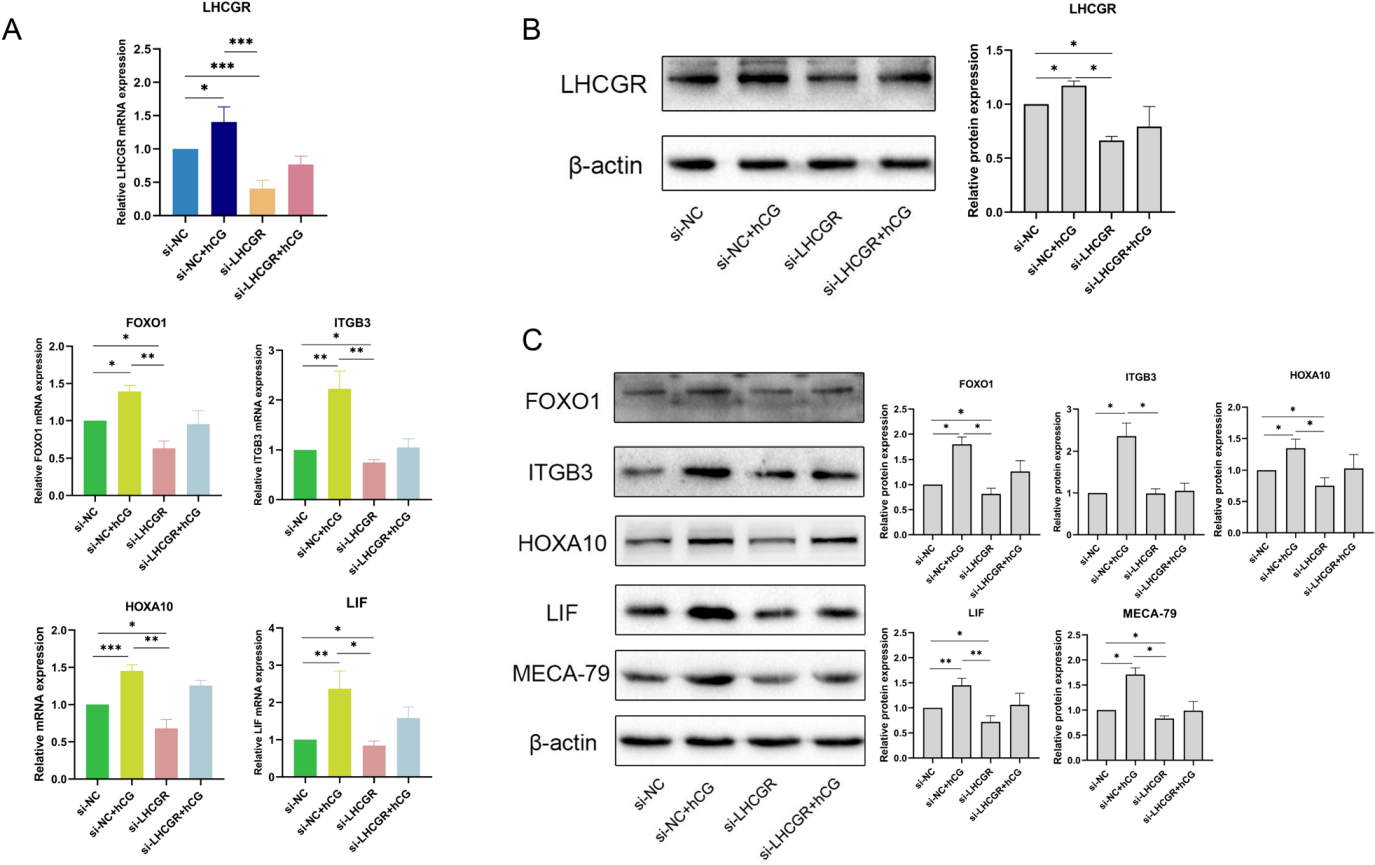
The expression levels of LHCGR, FOXO1, ITGB3 ,HOXA10, LIF and L-selectin ligand in ESCs after the knockdown of LHCGR. **A** The mRNA expression levels of LCGR, HOXA10, ITGB3, FOXO1 and LIF in ESCs after the knockdown of LHCGR. **B** The protein expression levels of LHCGR in ESCs were stimulated by hCG and knockdown of LHCGR. **C** The protein expression levels of FOXO1, ITGB3, HOXA10, LIF and L-selectin ligand in ESCs after the knockdown of LHCGR. The data were shown as mean ± SD (*n* = 3): *P < 0.05, **P < 0.01

To confirm whether LHCGR mediates the function of hCG in ESCs, siRNA-LHCGR was transfected into ESCs. As shown in Fig. 6A, 0.1 IU/mL hCG intervention enhanced the expression of LHCGR. After the transfection of siRNA-LHCGR, the level of LHCGR mRNA was decreased; intervention with 0.1 IU/mL hCG increased LHCGR, but the difference was not statistically significant (*p* > 0.05). The protein levels were also consistent (Fig. 6B), which suggested the unique activation and regulation characteristics of LHCGR. Subsequently, the expressions of HOXA10, ITGB3, FOXO1, LIF, and L-selectin ligands were determined, and the results showed that these were increased in the presence of hCG. However, the increased expressions of these molecules were inhibited after the transfection of siRNA-LHCGR (Fig. 6A and B). This finding implies that LHCGR is primarily responsible for the regulatory effect of hCG on endometrial receptivity molecules. In ESCs, hCG acts concertedly with LHCGR. Therefore, after knocking down LHCGR, this ability of hCG was reduced.

### hCG activated Autophagy in ESCs

**Fig. 7:**
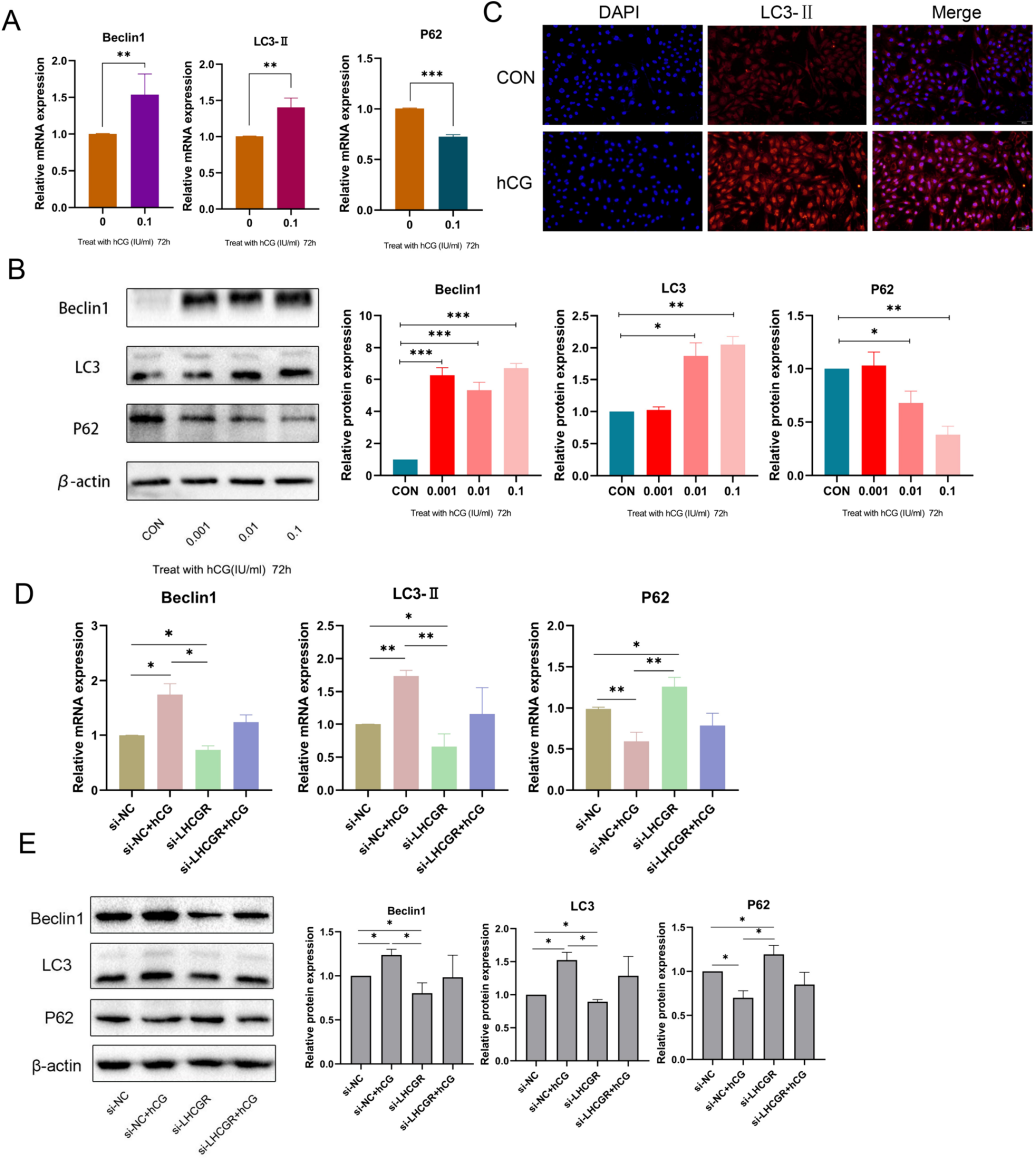
hCG intervention changes the level of autophagy in endometrial cells. **A** The mRNA expression levels of Beclin1, LC3-II and P62 in ESCs were stimulated by hCG for 72 h. **B** The protein expression levels of Beclin1, LC3 and P62 in ESCs were stimulated by hCG for 72 h. **C** Representative images of LC3-II in ESCs treated with hCG for 72 h. Scale: 50 μm. **D** The mRNA expression levels of Beclin1, LC3-II and P62 in ESCs after the knockdown of LHCGR. **E** The protein expression levels of Beclin1, LC3 and P62 in ESCs were stimulated by hCG and knockdown of LHCGR. Data were presented as mean ± SD (*n* = 3): *P < 0.05, **P < 0.01

The level of autophagy in endometrial cells is intricately linked to embryo implantation and decidualization [34, 35]. To determine whether hCG regulates autophagy in ESCs, the levels of autophagy-related molecules were estimated. The findings showed that hCG upregulated the expressions of LC3 and Beclin1 and downregulated the expression of P62 in ESCs compared with the control group (Fig. 7A and B), which indicates an increase in autophagy. To further assess the level of autophagy, the response of LC3-II to hCG was detected in ESCs using IF. The results alluded that LC3-II fluorescence was enhanced (Fig. 7C). This observation suggests that the ability of hCG to enhance endometrial receptivity may be associated with the increase in autophagy response. Furthermore, the effect of transfection of LHCGR on autophagy molecules was examined. As expected, the levels of Beclin1 and LC3 were lower in the si-LHCGR + hCG group than in the hCG group, whereas the level of P62 was higher (Fig. 7D and E). This finding signified that when LHCGR was turned off, the effect of hCG on activating autophagy was blocked. This result implies that LHCGR controls how gonadal hormones affect the autophagy function of ESCs.

### The level of apoptosis in ESCs increased after hCG intervention

**Fig. 8:**
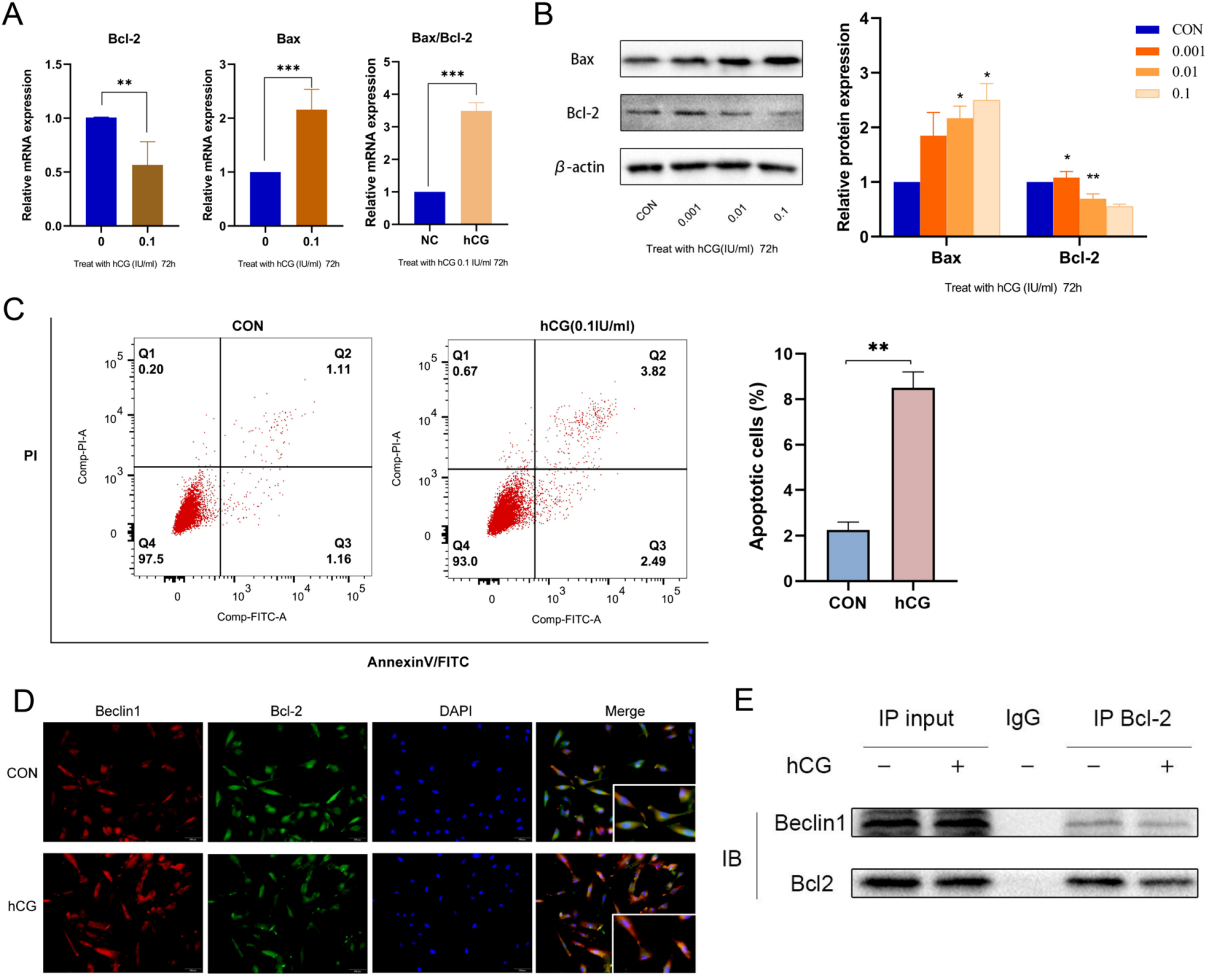
The level of endometrial cell apoptosis after hCG intervention. **A** The mRNA expression levels of Bax and Bcl-2 in ESCs stimulated with hCG for 72 h. **B** The protein expression levels of Bax and Bcl-2 in endometrial stromal cells after hCG stimulation of ESCs for 72 h. Data were presented as mean ± SD (*n* = 3): *P < 0.05, **P < 0.01, ***P < 0.001. **C** ESCs apoptotic cells were detected by flow cytometry after treatment with 0.1 IU/mL of hCG for 72 h. **D** Immunofluorescence staining was used to determine the localization of Beclin1 and Bcl-2, and DAPI solution for the nucleus staining.Scale: 50 μm. **E** Co-IP experiment was performed to confirm the effect of hCG on the interactions of Bcl-2 and Beclin1

The regulation of endometrial cell apoptosis plays a key role in tissue homeostasis and remodeling during the menstrual cycle, thereby preparing the endometrium for embryo implantation [36]. Favorable apoptosis is essential for decidualization in normal pregnancy [37]. Hence, the regulatory role of hCG in controlling apoptosis in endometrial decidual cells was investigated. RT-qPCR results demonstrated that compared with the control group, the expression of the proapoptotic gene Bax increased and that of the antiapoptotic gene Bcl-2 decreased in the hCG intervention group (Fig. 8A). Similarly, western blot analysis demonstrated that the expression of Bax was increased and that of Bcl-2 was significantly decreased in the hCG treatment group (Fig. 8B). Therefore, compared with the control group, the ratio of Bax/Bcl-2 increased significantly after hCG intervention (Fig. 8A). Furthermore, the changes in apoptosis were determined using flow cytometry, and the proportion of apoptotic cells is depicted in Fig. 8C. The proportions of late apoptotic or necrotic cells and early apoptotic cells are represented in Q2 and Q3 areas, respectively. The results showed that compared with the control group, the rate of apoptosis was increased after treatment with hCG (0.1 IU/mL, 72 h). These findings assert that hCG stimulation promotes endometrial receptivity and decidualization by altering the level of apoptosis.

Bcl-2 has been reported to prevent apoptosis by controlling the release of ΔΨm and cytochrome c, which are required to turn on caspase-9 [38]. Moreover, Bcl-2 induces autophagy by blocking Bcl-2–Beclin1 interaction and upregulating Beclin1 [39]. To determine the interaction between the two, the relationship between Beclin1 and Bcl-2 was studied. As illustrated in Fig. 8D, the co-localization of Beclin1 and Bcl-2 was observed using IF experiments. Further studies using co-IP confirmed this finding, indicating that Beclin1 interacts with Bcl-2. Figure 8E shows that Bcl-2 IP decreased Beclin1 in ESCs and that Bcl-2 pulled down Beclin1 more in control cells than in hCG-treated cells. This finding alludes that hCG treatment prevented Bcl-2 and Beclin1 from interacting with each other and starting an autophagy response.

### The phosphorylation levels of ERK and mTOR in ESCs increased after hCG intervention

**Fig. 9:**
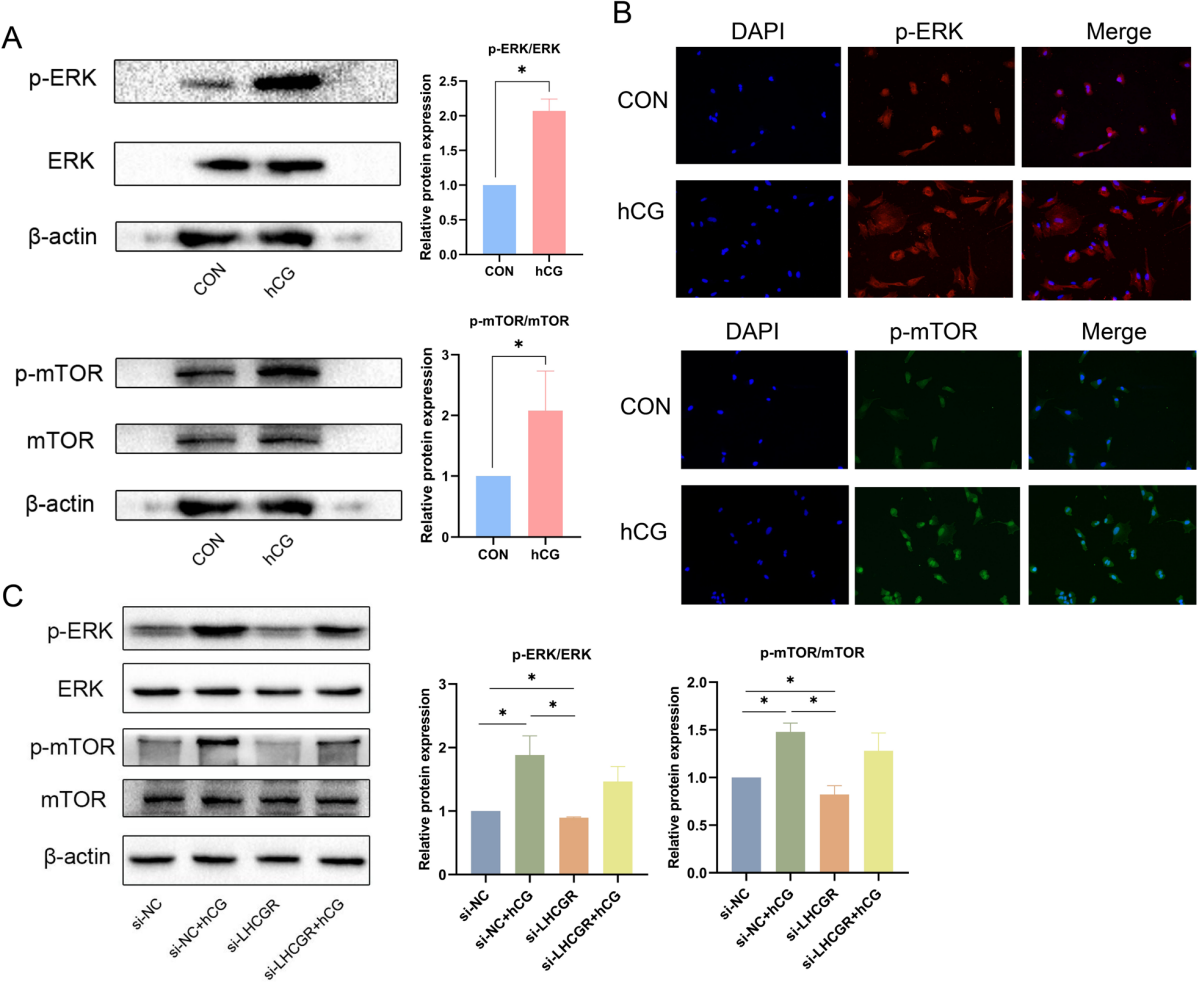
Changes of ERK and mTOR phosphorylation levels in endometrial cells after hCG intervention. **A** The protein expression levels of p-mTOR and p-ERK1/2 in endometrial stromal cells after hCG stimulation of ESCs for 72 h. **B** Representative images of p-mTOR and p-ERK in ESCs treated with hCG for 72 h. Scale: 50 μm. **C** The protein expression levels of p-ERK1/2 and p-mTOR in ESCs after knockdown of LHCGR. Data were presented as mean ± SD (*n* = 3): *P < 0.05

Endometrial receptivity is a complex process that can be regulated by various signaling pathways, such as ERK1/2 and mTOR. These are currently considered to be two signaling pathways closely related to autophagy. ERK activation leads to apoptosis and autophagy induction, and mTOR is also known as the main regulator of autophagy. Subsequently, to discern the biological role of hCG/LHCGR, immunoblotting was used to examine the phosphorylation of ERK1/2 and mTOR in ESCs cultured in the presence of 0.1 IU hCG. The findings indicated that compared with the control group, hCG intervention increased the levels of phosphorylated ERK1/2 (p-ERK1/2) and mTOR (p-mTOR) in ESCs (Fig. 9A). Moreover, IF results indicated that hCG stimulation enhanced the expressions of p-ERK and p-mTOR (Fig. 9B). Therefore, ERK and mTOR signaling pathways may play a pertinent role in the way in which hCG triggers autophagy and establishes endometrial receptivity. Finally, phosphorylation in the presence of siRNA-LHCGR was verified. The results showed that LHCGR knockdown attenuated the stimulatory effect of hCG on p-ERK and p-mTOR (Fig. 9C). In summary, the function of hCG in ESCs is mediated by LHCGR and is linked to the activation of ERK1/2 and mTOR pathways in the cells.

## Discussion

The process of embryo implantation comprises three successive stages, namely, localization, adhesion, and invasion, which are regulated by a myriad of cells and factors [40]. Their interactions construct the complex regulatory network necessary for successful embryo implantation [41]. However, currently we do not have a complete understanding of this regulatory network. The findings from this study indicate that endometrial receptivity was compromised in women with RIF and that the expression levels of LHCGR and the process of autophagy in endometrial tissues were reduced. Nonetheless, when ESCs were treated with hCG, LHCGR was activated, which mediated intracellular functionality, modulated ESC autophagy and apoptosis by inducing the phosphorylation of ERK1/2 and mTOR, and influenced the expression of endometrial receptivity factors.

Embryo implantation in the maternal endometrium is an important stage in the reproductive process, and implantation failure is one of the main challenges in assisted reproductive technology [42]. hCG, a member of the glycoprotein hormone family, is a distinct signal marker of the embryo. The secretion of hCG by blastocysts prior to the implantation of the embryo in the maternal endometrium suggests a potential association with LHCGR expressed in the endometrium [43]. The findings from this study confirm this hypothesis. Our observations indicate that after hCG treatment, IF and gene and protein expressions of LHCGR were significantly enhanced in ESCs, which were weakened after LHCGR knockdown. This suggests that LHCGR holds physiological significance, essentially implying that the cellular functionality of hCG is accomplished via LHCGR. This receptor has previously been identified in various extragonadal tissues and extensively across the female reproductive tract [13, 44–47]. These insights validate the presence of functionally active LHCGR in the human endometrium. Several studies have established the role of LHCGR in decidualization, such as in promoting prolactin secretion [15, 48–50]. Our results signify that the expression level of LHCGR is decreased in the endometrial tissue of patients with RIF and the decidualization ability is reduced, which implies that the functionality of hCG and/or LH in endometrial physiology may be compromised to different extents. Previous studies have found that female gonadal development, spermatogenesis, and uterine development are associated with the LHCGR and CYP19A1 genes [51]. Sacchi et al. also found that LHCGR was expressed in the endometrium of women during the menstrual cycle. And its stimulation with gonadotropins can promote intracellular cAMP accumulation, up-regulate the expression of steroidogenic acute regulatory protein (StAR) and 3β-hydroxysteroid dehydrogenase/Δ5 − 4 isomerase (3β-HSD), and increase the synthesis of local progesterone in mammalian endometrium [44]. On the one hand, these data suggest that hCG and LHCGR may play a role in establishing endometrial receptivity by regulating endometrial metabolism and growth. On the other hand, it shows that hCG supplementation may enhance the role of this hormone in the endometrium by increasing the expression of LHCGR, thereby improving endometrial function in patients with RIF. However, our sample size was relatively small, which limits the breadth of our conclusions. Further in-depth studies with a larger sample size are necessary to elucidate the exact role of LHCGR in the complex signal transduction network that regulates the physiological processes of decidualization and implantation as well as the associated reproductive results. Moreover, the potential diagnostic and therapeutic effects of LHCGR on endometrial function should be investigated.

Despite the rapid development and widespread clinical application of IVF in patients with infertility over the past few decades, RIF remains a major challenge in human reproduction [52]. The etiology of RIF is complex, and one of the most important reasons is embryo–endometrial asynchrony, shift in the window of implantation (WOI), and impaired endometrial receptivity. The establishment and coordination of endometrial receptivity are mainly responsible by the estrogen and progesterone [53]. Several adhesion molecules, cytokines, growth factors, and lipid mediators, such as IGFBP, PRL, HOXA10, WNT, and LIF, regulate endometrial receptivity by participating in complex signal transduction and angiogenesis [54]. Joshi et al. showed that compared with normal women of childbearing age, the expression levels of these factors were significantly lower in the endometrium of patients with infertility [55]. This reduced expression affected trophoblast invasion during endometrial decidualization and early placental formation, thus promoting the development of RIF [56]. Our findings suggest that HOXA10 and ITGB3 levels are low in the uterine tissue of women with RIF. The HOXA gene cluster plays a vital role in morphological alterations in the female reproductive tract and cyclic variations in the endometrium [57]. HOXA is considered to be involved in implantation function [58]. Female mice lacking HOXA10 have been reported to experience spontaneous abortion and infertility owing to implantation and decidualization failure [59]. HOXA10 expression is associated with variations in the expressions of downstream target genes, including ITGB3, which are biomarkers of endometrial receptivity [60]. The expression of ITGB3 increases in the mid-secretory phase, which corresponds to WOI. Hence, ITGB3 is also regarded as a decidualization marker in ESCs [61]. In our study, we established a mechanism where the protein and mRNA levels of HOXA10 and ITGB3 increased significantly in ESCs treated with 0.1 IU/mL hCG for 72 h. This finding implies that hCG may promote decidualization and enhance endometrial receptivity by increasing the expression levels of HOXA10 and ITGB3 in ESCs. Furthermore, the knockdown of LHCGR inhibited this change. This observation signifies that in ESCs, hCG mainly functions via LHCGR, which may also be one of the reasons for the decreased endometrial receptivity in patients with RIF. The decreased expression of LHCGR in the endometrial tissue appears to affect the functional effect of hCG. Furthermore, hCG can modulate the expressions of LIF, L-selectin ligand, and FOXO1. LIF and L-selectin ligands play a major role in initial embryo attachment and adhesion. These molecules can mediate pinopodes to participate in embryo–endometrial interaction and are important local media for successful embryo implantation in the endometrium [62, 63]. FOXO1 is considered to be a key regulator of endometrial receptivity in vivo [64] and is involved in endometrial remodeling during the menstrual cycle and early pregnancy, regulating the transcription of decidual prolactin (PRL) and insulin-like growth factor-binding protein 1 (IGFBP-1) genes [65]. This might constitute an alternative mechanism by which hCG promotes embryo implantation.

Recent studies have emphasized the role of autophagy in regulating the development of reproductive organs and germ cells [66, 67]. Particularly, endometrial autophagy is indispensable for maintaining endometrial homeostasis, including menstruation, decidualization [30] and WOI [33, 68, 69]. In our study, the expression levels of LC3 and P62 in the endometrial tissue of patients with RIF were assessed. LC3, a key structural component of autophagic vesicles, promotes the formation of autophagosomes [70]. P62 is another crucial marker used to measure autophagy impairment [71, 72]. Our findings showed a decrease in LC3 levels and an increase in P62 protein levels in the endometrium of patients with RIF and the autophagy reaction was reduced. A previous study observed that autophagy is activated during the decidualization of ESCs and that inhibiting this process reduces the levels of PRL and IGFBP1 and results in decidualization impairment [73]. Furthermore, a single-cell transcriptome sequencing of RIF showed that several genes for endometrial receptivity (such as LIF, IL6ST, and ITGA3) and autophagy (such as ATG9B and APOL1) were downregulated in the endometrial cells of patients with RIF [52]. Our results support the notion that endometrial autophagy is important for embryo implantation during WOI and that one reason for the failure of embryo implantation in patients with RIF is the improper functioning of endometrial autophagy. Therefore, further research is needed to focus on their value in RIF prediction and treatment. For example, autophagy activators may be a potential therapeutic option for women with recurrent miscarriage or infertility.

In our cell experiments, hCG stimulation increased the expressions of Beclin1 and LC3II/I in ESCs and decreased the expression of P62, indicating significant autophagy activation, which could help improve endometrial condition and support embryo implantation. Moreover, this effect decreased when si-LHCGR was added, again emphasizing that the function of hCG depends on the presence of receptors. Additionally, autophagy has been shown to respond to intricate alterations in gene expression in ESCs during the establishment of WOI [73] and to regulate the release of proapoptotic factors [74, 75]. This finding is aligned with the results of our study, suggesting that hCG can influence endometrial receptivity and decidualization by augmenting autophagy and apoptosis. These findings provide new insights into solving reproductive problems associated with implantation failure and strengthen the clinical application of hCG in patients with infertility, serving as a key auxiliary method for enhancing implantation and pregnancy outcomes in women with RIF. However, a single detection method was used in our study to determine the protein levels; hence, it is necessary to further explore the specific mechanisms of autophagy in future studies.

During a typical pregnancy, moderate levels of apoptosis are considered crucial for establishing endometrial receptivity and facilitating decidualization [76–80]. The endometrium creates an intrauterine environment that is conducive to embryo implantation via changes in proliferation and apoptosis to adapt to embryo implantation and development [81]. When apoptosis is decreased, it could lead to excessive endometrial thickening and dysfunction, thereby affecting its receptivity to embryos [82]. In this study, hCG treatment upregulated the expression of the apoptosis-related protein Bax and downregulated the expression of Bcl-2, and the results of flow cytometry also showed that apoptosis occurred. This finding suggests that hCG might enhance endometrial receptivity by regulating apoptosis. Nevertheless, the role of apoptosis in pregnancy is complex, and excessive apoptosis might also be detrimental [83]. Therefore, understanding how hCG precisely modulates apoptosis both temporally and spatially is essential for unraveling its role in embryo implantation.

Increasing evidence has confirmed the presence of a cooperative relationship between apoptosis and autophagy [84–86]. These are two important processes with complex protein networks. Beclin1, an autophagy molecule, possesses a BH3 domain. Bcl-2 can bind to Beclin1 via the BH3 structural groove and change the way in which it works [87]. When Beclin1 and Bcl-2 work concertedly, they lower the amount of free Beclin1 in cells, which makes the type III PI3K complex less active, thereby retarding autophagy; on the contrary, autophagy is activated. In this study, Beclin1 and Bcl-2 were co-localized. Co-IP showed that Bcl-2 could interact with Beclin1. hCG treatment reduced the expression of Bcl-2 and weakened the interaction between Bcl-2 and Beclin1, as a result of which Beclin1 was released, which in turn triggered the autophagy cascade.

In addition, the impact of hCG on the ERK and mTOR signaling pathways was investigated. Two key hyperactivated signaling pathways, PI3K/AKT/mTOR and RAS/RAF/MEK/ERK, are implicated in endometrial receptivity and ESC differentiation [88]. During early embryo implantation, the immunoreactivity of mTOR within the uterus is elevated [89]. Decreased p-ERK expression leads to disordered decidualization and an increased abortion rate [90], which implies that both ERK and mTOR play pivotal roles in embryo implantation and decidualization. We also observed that the levels of p-ERK1/2 and p-mTOR in ESCs increased after hCG stimulation and decreased after LHCGR knockdown. This suggests that the role of hCG/LHCGR may be linked to the activation of the ERK-mTOR pathway. Moreover, ERK and mTOR are closely associated with autophagy [91]. ERK phosphorylation aids in promoting the increase of LC3 and Beclin1 and inducing autophagy and apoptosis [92], which is consistent with our conclusion. As an autophagy controller, mTOR is a negative regulator of autophagy [93]. In our study, hCG stimulation increased mTOR, which may be related to its reactivation during the regulation of autophagy by hCG. A study recently identified that if the activation time for autophagy is long, macromolecules are degraded in autolysosomes, which allows amino acids to escape into the cytosol via efflux transporters. This movement of amino acids turns on mTOR again in that area and starts autophagic lysosomal recombination [94].

**Fig. 10:**
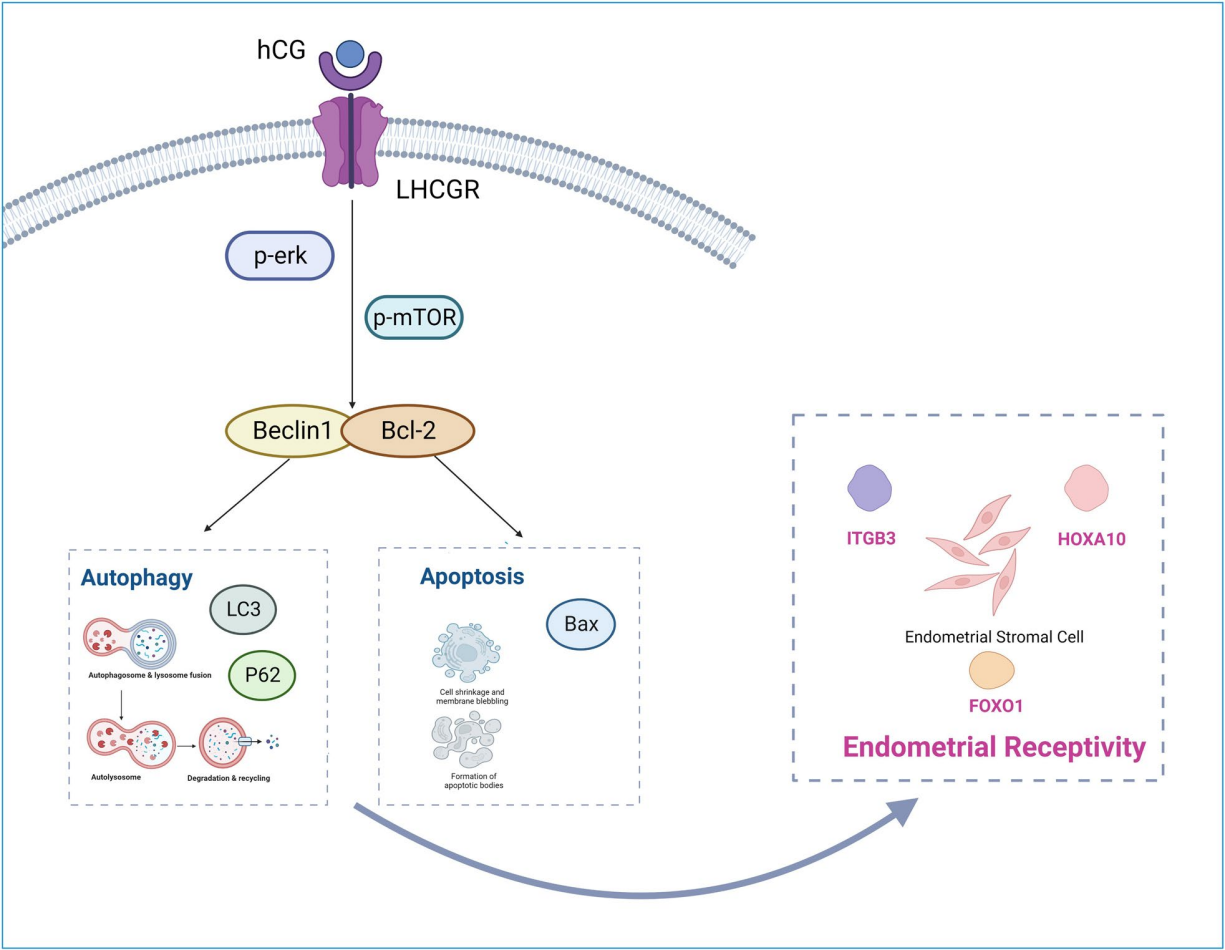
The schematic presentation of the roles of human chorionic gonadotropin in autophagy and apoptosis regulation in endometrial stromal cells

In conclusion, our study has demonstrated that LHCGR-mediated hCG enhances the expressions and functions of HOXA10, ITGB3, FOXO1, LIF, and L-selectin ligands, all of which are involved in embryo implantation. This is achieved by regulating the levels of autophagy and apoptosis, thereby improving endometrial receptivity and facilitating decidualization of stromal cells and embryo implantation. These molecular alterations are likely to be mediated via the mTOR and ERK1/2 signaling pathways (Fig. 10). However, our research does have certain limitations. Further studies are required to understand the specific roles and interactions of these signaling pathways as well as the precise role of hCG in these processes. Future investigations should aim to unravel the detailed mechanisms underlying these pathways. Furthermore, potential treatment strategies targeting these pathways must be devised to improve women’s fertility. Despite these limitations, our findings have the potential to significantly contribute to our understanding of the complex interplay among hCG, endometrial receptivity, and embryo implantation. This could pave the way for the development of innovative therapeutic strategies to address RIF and other conditions related to female infertility.

### Supplementary Information

**Supplementary Material 1.**

## Data Availability

No datasets were generated or analysed during the current study.

## References

[1] Evans J (2016). Fertile ground: human endometrial programming and lessons in health and disease. Nat Reviews Endocrinol.

[2] Altmäe S (2017). Meta-signature of human endometrial receptivity: a meta-analysis and validation study of transcriptomic biomarkers. Sci Rep.

[3] Shekibi M (2022). MicroRNAs in the regulation of endometrial receptivity for embryo implantation. Int J Mol Sci.

[4] Deryabin P (2021). All-in-one genetic tool assessing endometrial receptivity for personalized screening of female sex steroid hormones. Front Cell Dev Biol.

[5] Jiang L, et al. Single-cell RNA-sequencing reveals interactions between endometrial stromal cells, epithelial cells, and lymphocytes during mouse embryo implantation. Int J Mol Sci. 2022;24(1): 213. 10.3390/ijms24010213 PMC9820401 36613656

[6] Jurisicova A (1999). Variability in the expression of trophectodermal markers beta-human chorionic gonadotrophin, human leukocyte antigen-G and pregnancy specific beta-1 glycoprotein by the human blastocyst. Hum Reprod (Oxford England).

[7] Ramu S (2011). Human chorionic gonadotropin from day 2 spent embryo culture media and its relationship to embryo development. Fertil Steril.

[8] d’Hauterive SP (2022). Human chorionic gonadotropin and early embryogenesis. Rev Int J Mol Sci.

[9] Tapia-Pizarro A (2017). hCG activates Epac-Erk1/2 signaling regulating progesterone receptor expression and function in human endometrial stromal cells. Mol Hum Reprod.

[10] Gao M (2019). Intrauterine injection of human chorionic gonadotropin before embryo transfer can improve in vitro fertilization-embryo transfer outcomes: a meta-analysis of randomized controlled trials. Fertil Steril.

[11] Licht P (1998). Novel insights into human endometrial paracrinology and embryo-maternal communication by intrauterine microdialysis. Hum Reprod Update.

[12] Srivastava A (2013). Profiles of cytokines secreted by isolated human endometrial cells under the influence of chorionic gonadotropin during the window of embryo implantation. Reprod Biol Endocrinol.

[13] Licht P (2003). Evidence for cycle-dependent expression of full-length human chorionic gonadotropin/luteinizing hormone receptor mRNA in human endometrium and decidua. Fertil Steril.

[14] Sha J (2017). Alteration of Th17 and Foxp3(+) regulatory T cells in patients with unexplained recurrent spontaneous abortion before and after the therapy of hCG combined with immunoglobulin. Exp Ther Med.

[15] Mann ON (2022). Expression and function of the luteinizing hormone choriogonadotropin receptor in human endometrial stromal cells. Sci Rep.

[16] Xie YB (1990). Extracellular domain of lutropin/choriogonadotropin receptor expressed in transfected cells binds choriogonadotropin with high affinity. J Biol Chem.

[17] Casarini L (2018). Two hormones for one receptor: evolution, biochemistry, actions, and pathophysiology of LH and hCG. Endocr Rev.

[18] Zhong Y (2019). Association of hCG and LHCGR expression patterns with clinicopathological parameters in ovarian cancer. Pathol Res Pract.

[19] Filicori M (2005). Novel concepts of human chorionic gonadotropin: reproductive system interactions and potential in the management of infertility. Fertil Steril.

[20] Roskoski R (2012). ERK1/2 MAP kinases: structure, function, and regulation. Pharmacol Res.

[21] Banerjee P (2010). Endometrial responses to embryonic signals in the primate. Int J Dev Biol.

[22] Liu H (2020). Epigenetic modifications working in the decidualization and endometrial receptivity. Cell Mol Life Sci.

[23] Ma L (2005). Phosphorylation and functional inactivation of TSC2 by Erk implications for tuberous sclerosis and cancer pathogenesis. Cell.

[24] Zhan X (2021). Dexamethasone may inhibit placental growth by blocking glucocorticoid receptors via phosphatidylinositol 3-kinase/AKT/mammalian target of rapamycin and reactive oxygen species/AMP-activated protein kinase signalling pathways in human placental JEG-3 cells. Reprod Fertil Dev.

[25] Roberti SL (2018). Critical role of mTOR, PPARγ and PPARδ signaling in regulating early pregnancy decidual function, embryo viability and feto-placental growth. Mol Hum Reprod.

[26] Dimasuay KG (2016). Placental responses to changes in the maternal environment determine fetal growth. Front Physiol.

[27] Hesam Shariati MB (2019). The effect of fludrocortisone on the uterine receptivity partially mediated by ERK1/2-mTOR pathway. J Cell Physiol.

[28] Shariati MBH (2019). Administration of dexamethasone disrupts endometrial receptivity by alteration of expression of miRNA 223, 200a, LIF, Muc1, SGK1, and ENaC via the ERK1/2-mTOR pathway. J Cell Physiol.

[29] Yang S (2019). Role of endometrial autophagy in physiological and pathophysiological processes. J Cancer.

[30] Choi J (2012). The role of autophagy in human endometrium. Biol Reprod.

[31] Choi S (2014). Suppression of autophagic activation in the mouse uterus by estrogen and progesterone. J Endocrinol.

[32] Lu H (2021). Rapamycin prevents spontaneous abortion by triggering decidual stromal cell autophagy-mediated NK cell residence. Autophagy.

[33] Su Y (2020). Endometrial autophagy is essential for embryo implantation during early pregnancy. J Mol Med.

[34] Oestreich AK (2020). The autophagy protein, FIP200 (RB1CC1) mediates progesterone responses governing uterine receptivity and decidualization†. Biol Reprod.

[35] Oestreich AK (2020). The autophagy gene Atg16L1 is necessary for endometrial decidualization. Endocrinology.

[36] Fluhr H (2013). Constitutive activity of Erk1/2 and NF-κB protects human endometrial stromal cells from death receptor-mediated apoptosis. Reprod Biol.

[37] Han M (2014). sHLA-G involved in the apoptosis of decidual natural killer cells following toxoplasma gondii infection. Inflammation.

[38] Lindsay J (2011). Bcl-2 proteins and mitochondria–specificity in membrane targeting for death. Biochim Biophys Acta.

[39] Lian J (2011). A natural BH3 mimetic induces autophagy in apoptosis-resistant prostate cancer via modulating bcl-2-Beclin1 interaction at endoplasmic reticulum. Cell Death Differ.

[40] Makrigiannakis A (2007). Mechanisms of implantation. Reprod Biomed Online.

[41] de Ruijter-Villani M (2015). The role of conceptus-maternal signalling in the acquisition of uterine receptivity to implantation in mammals. Reprod Domest Anim.

[42] Hammadeh ME (2011). Assisted hatching in assisted reproduction: a state of the art. J Assist Reprod Genet.

[43] Zhu M (2020). Human chorionic gonadotropin improves endometrial receptivity by increasing the expression of homeobox A10. Mol Hum Reprod.

[44] Sacchi S (2018). Evidence for expression and functionality of FSH and LH/hCG receptors in human endometrium. J Assist Reprod Genet.

[45] Rahman NA (2009). Recent progress in luteinizing hormone/human chorionic gonadotrophin hormone research. Mol Hum Reprod.

[46] Reshef E (1990). The presence of gonadotropin receptors in nonpregnant human uterus, human placenta, fetal membranes, and decidua. J Clin Endocrinol Metab.

[47] Popovici RM (2000). Discovery of new inducible genes in in vitro decidualized human endometrial stromal cells using microarray technology. Endocrinology.

[48] Han SW (1999). Treatment of human endometrial stromal cells with chorionic gonadotropin promotes their morphological and functional differentiation into decidua. Mol Cell Endocrinol.

[49] Kasahara K (2001). The role of human chorionic gonadotropin on decidualization of endometrial stromal cells in vitro. J Clin Endocrinol Metab.

[50] Kajihara T (2011). Human chorionic gonadotropin confers resistance to oxidative stress-induced apoptosis in decidualizing human endometrial stromal cells. Fertil Steril.

[51] Wang Z (2021). Berberine improves ovulation and endometrial receptivity in polycystic ovary syndrome. Phytomedicine.

[52] Ghasemnejad-Berenji H (2018). Immunomodulatory effects of hydroxychloroquine on Th1/Th2 balance in women with repeated implantation failure. Biomed Pharmacother.

[53] Lessey BA (2019). What exactly is endometrial receptivity?. Fertil Steril.

[54] Lai ZZ (2022). Single-cell transcriptome profiling of the human endometrium of patients with recurrent implantation failure. Theranostics.

[55] Joshi NR (2021). Genetic and epigenetic changes in the eutopic endometrium of women with endometriosis: association with decreased endometrial αvβ3 integrin expression. Mol Hum Reprod.

[56] Hu C (2024). Endometrial BMP2 deficiency impairs ITGB3-mediated trophoblast invasion in women with repeated implantation failure. Endocrinology.

[57] Du H (2015). The role of hox genes in female reproductive tract development, adult function, and fertility. Cold Spring Harb Perspect Med.

[58] Bi Y (2022). HOXA10 improves endometrial receptivity by upregulating E-cadherin†. Biol Reprod.

[59] Qian K (2005). Differentiation of endometrial stromal cells in vitro: down-regulation of suppression of the cell cycle inhibitor p57 by HOXA10?. Mol Hum Reprod.

[60] Ekanayake DL (2022). The roles and expression of HOXA/Hoxa10 gene: a prospective marker of mammalian female fertility?. Reprod Biol.

[61] Peng Y (2021). Scribble downregulation in adenomyosis compromises endometrial stromal decidualization by decreasing FOXO1 expression. Hum Reprod (Oxford England).

[62] Basatvat S (2021). Potential innate immunity-related markers of endometrial receptivity and recurrent implantation failure (RIF). Reprod Biol.

[63] Mokhtar MH (2020). Testosterone decreases the number of implanting embryos, expression of Pinopode and L-selectin ligand (MECA-79) in the Endometrium of early pregnant rats. Int J Environ Res Public Health.

[64] Vasquez YM (2018). FOXO1 regulates uterine epithelial integrity and progesterone receptor expression critical for embryo implantation. PLoS Genet.

[65] Adiguzel D (2021). FoxO1 is a cell-specific core transcription factor for endometrial remodeling and homeostasis during menstrual cycle and early pregnancy. Hum Reprod Update.

[66] Peters AE (2020). Autophagy in female fertility: a role in oxidative stress and aging. Antioxid Redox Signal.

[67] Zhu Y (2019). Autophagy in male reproduction. Syst Biol Reprod Med.

[68] Lee JE (2011). Autophagy regulates embryonic survival during delayed implantation. Endocrinology.

[69] Kim SM (2017). A review of mechanisms of implantation. Dev Reprod.

[70] Parzych KR (2014). An overview of autophagy: morphology, mechanism, and regulation. Antioxid Redox Signal.

[71] Lamark T (2017). Regulation of selective autophagy: the p62/SQSTM1 paradigm. Essays Biochem.

[72] Zhu Y (2022). Autophagy markers are dysregulated in the endometrial tissues of patients with unexplained repeated implantation failure. Mol Reprod Dev.

[73] Mestre Citrinovitz AC (2019). Decreased Autophagy impairs decidualization of human endometrial stromal cells: a role for ATG Proteins in endometrial physiology. Int J Mol Sci.

[74] Wu F (2015). Oxidative stress in placenta: health and diseases. Biomed Res Int.

[75] Kroemer G (2010). Autophagy and the integrated stress response. Mol Cell.

[76] Long J (2019). FOXO3a is essential for murine endometrial decidualization through cell apoptosis during early pregnancy. J Cell Physiol.

[77] Boeddeker SJ (2015). Decidualization and syndecan-1 knock down sensitize endometrial stromal cells to apoptosis induced by embryonic stimuli. PLoS One.

[78] Leno-Durán E (2014). Human decidual stromal cells secrete soluble pro-apoptotic factors during decidualization in a cAMP-dependent manner. Hum Reprod (Oxford England).

[79] Wei P (2003). Molsidomine and N-omega-nitro-L-arginine methyl ester inhibit implantation and apoptosis in mouse endometrium. Acta Pharmacol Sin.

[80] Yi T (2019). Benzo(a)pyrene inhibits endometrial cell apoptosis in early pregnant mice via the WNT5A pathway. J Cell Physiol.

[81] Pan H (2006). Progesterone blocks estrogen-induced DNA synthesis through the inhibition of replication licensing. Proc Natl Acad Sci USA.

[82] Yan L (2012). Expression of apoptosis-related genes in the endometrium of polycystic ovary syndrome patients during the window of implantation. Gene.

[83] Yu D (2020). Exposure to acrylamide inhibits uterine decidualization via suppression of cyclin D3/p21 and apoptosis in mice. J Hazard Mater.

[84] He C (2013). Dissociation of bcl-2-Beclin1 complex by activated AMPK enhances cardiac autophagy and protects against cardiomyocyte apoptosis in diabetes. Diabetes.

[85] Salminen A (2013). Beclin 1 interactome controls the crosstalk between apoptosis, autophagy and inflammasome activation: impact on the aging process. Ageing Res Rev.

[86] Li Z (2019). The interaction of Atg4B and Bcl-2 plays an important role in Cd-induced crosstalk between apoptosis and autophagy through disassociation of bcl-2-Beclin1 in A549 cells. Free Radic Biol Med.

[87] Oberstein A (2007). Crystal structure of the Bcl-XL-Beclin 1 peptide complex: Beclin 1 is a novel BH3-only protein. J Biol Chem.

[88] Lee CH (2013). Extracellular signal-regulated kinase 1/2 signaling pathway is required for endometrial decidualization in mice and human. PLoS One.

[89] Ekizceli G (2017). Assessment of mTOR pathway molecules during implantation in rats. Biotech Histochem.

[90] Zhang S (2023). Role of kisspeptin in decidualization and unexplained recurrent spontaneous abortion via the ERK1/2 signalling pathway. Placenta.

[91] Wang B (2021). Enterovirus 71 induces autophagy in mice via mTOR inhibition and ERK pathway activation. Life Sci.

[92] Wang J (2020). Morusin induces apoptosis and autophagy via JNK, ERK and PI3K/Akt signaling in human lung carcinoma cells. Chem Biol Interact.

[93] Panwar V (2023). Multifaceted role of mTOR (mammalian target of rapamycin) signaling pathway in human health and disease. Signal Transduct Target Ther.

[94] Nanayakkara R (2023). Autophagic lysosome reformation in health and disease. Autophagy.

