# Supplementary figures and images for: Enhancing endometrial receptivity: the roles of human chorionic gonadotropin in autophagy and apoptosis regulation in endometrial stromal cells
Source: Reprod Biol Endocrinol. 2024 Apr 4;22:37. doi: 10.1186/s12958-024-01205-x (PMC10993617; doi:10.1186/s12958-024-01205-x)

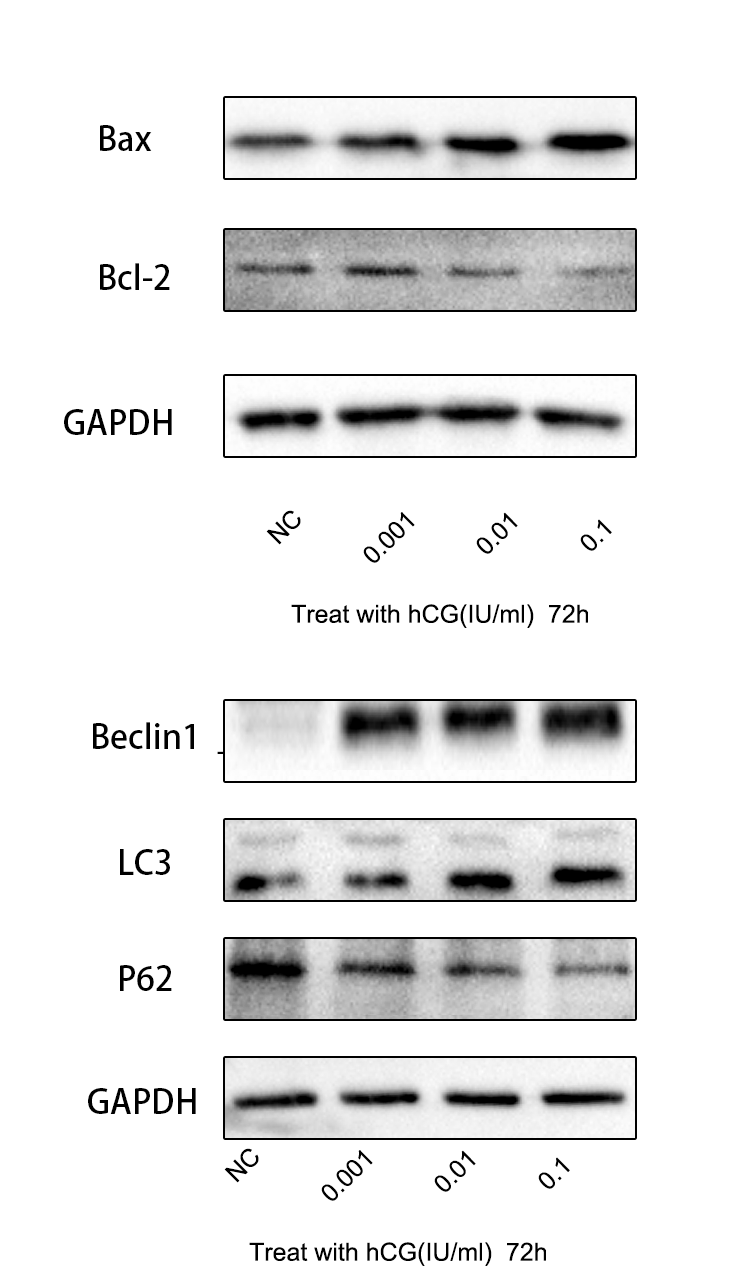

Supplement: Supplementary file 1 — Supplementary Material 1. [file 12958_2024_1205_MOESM1_ESM.zip › WESTERN/autophagy+apoptosis-1-ESCs.tif]

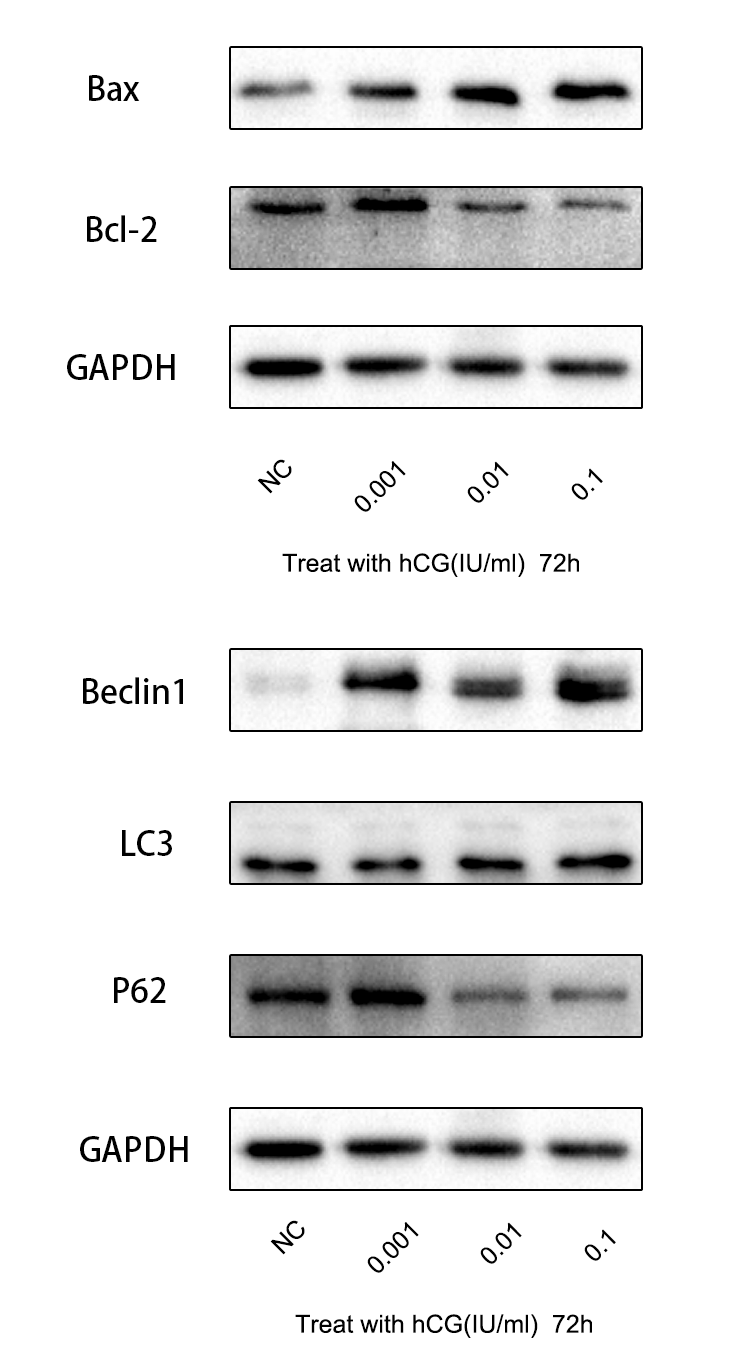

Supplement: Supplementary file 1 — Supplementary Material 1. [file 12958_2024_1205_MOESM1_ESM.zip › WESTERN/autophagy+apoptosis-2-ESCs.tif]

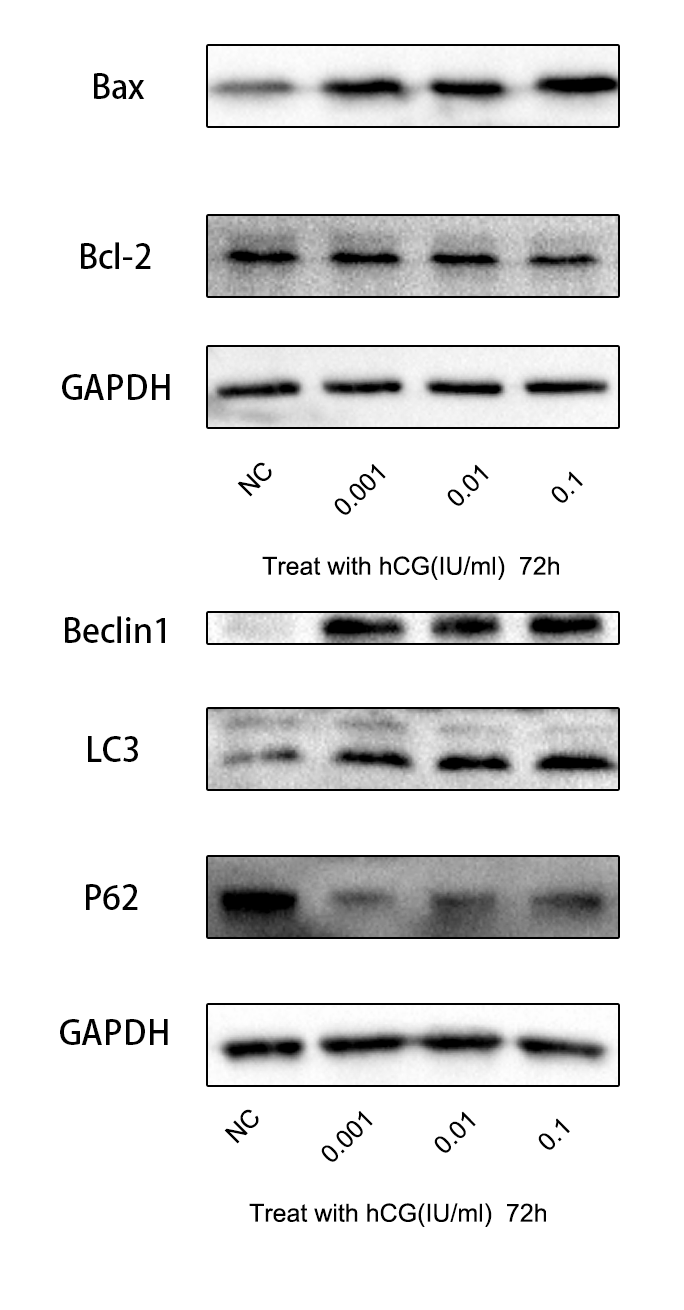

Supplement: Supplementary file 1 — Supplementary Material 1. [file 12958_2024_1205_MOESM1_ESM.zip › WESTERN/autophagy+apoptosis-3--ESCs.tif]

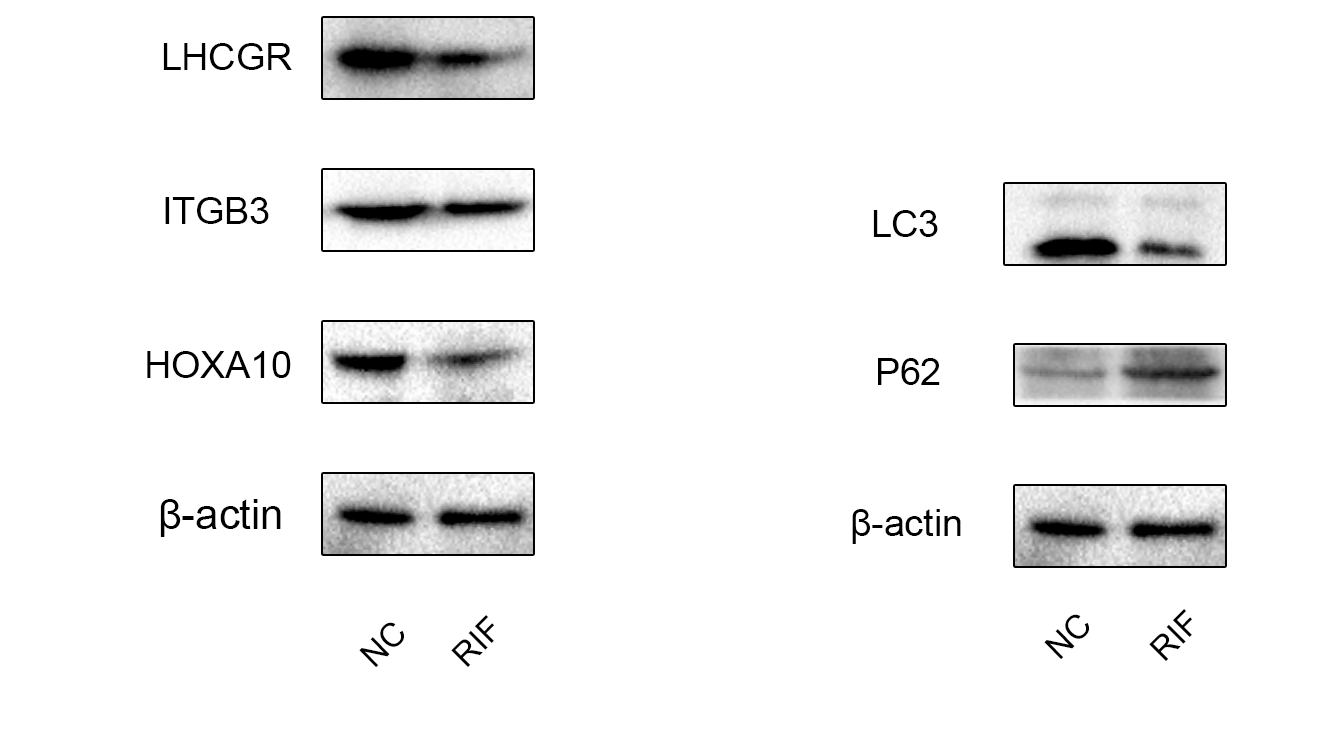

Supplement: Supplementary file 1 — Supplementary Material 1. [file 12958_2024_1205_MOESM1_ESM.zip › WESTERN/endometrial tissue-1.tif]

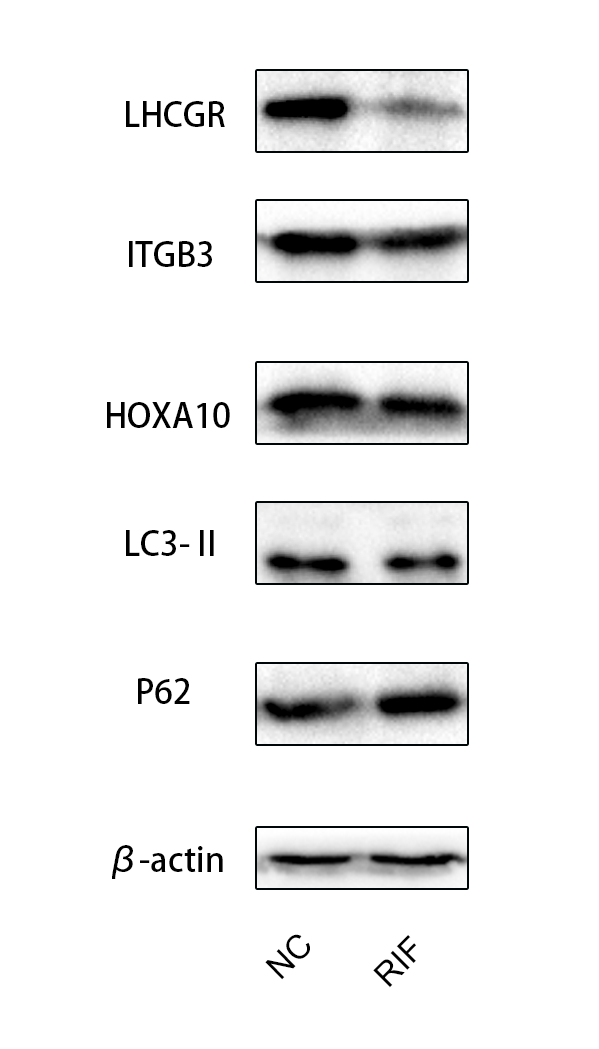

Supplement: Supplementary file 1 — Supplementary Material 1. [file 12958_2024_1205_MOESM1_ESM.zip › WESTERN/endometrial tissue-2.tif]

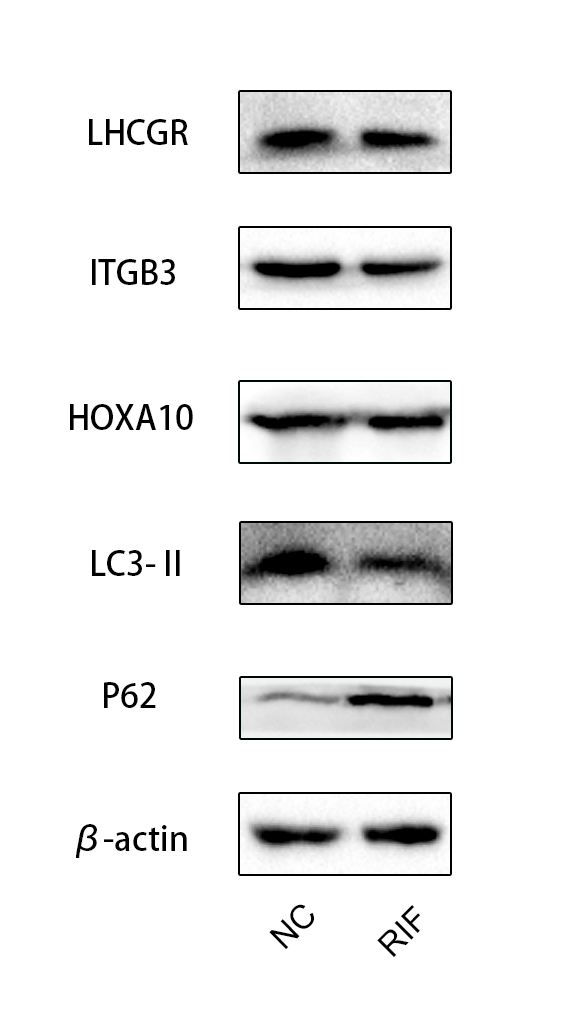

Supplement: Supplementary file 1 — Supplementary Material 1. [file 12958_2024_1205_MOESM1_ESM.zip › WESTERN/endometrial tissue-3.tif]

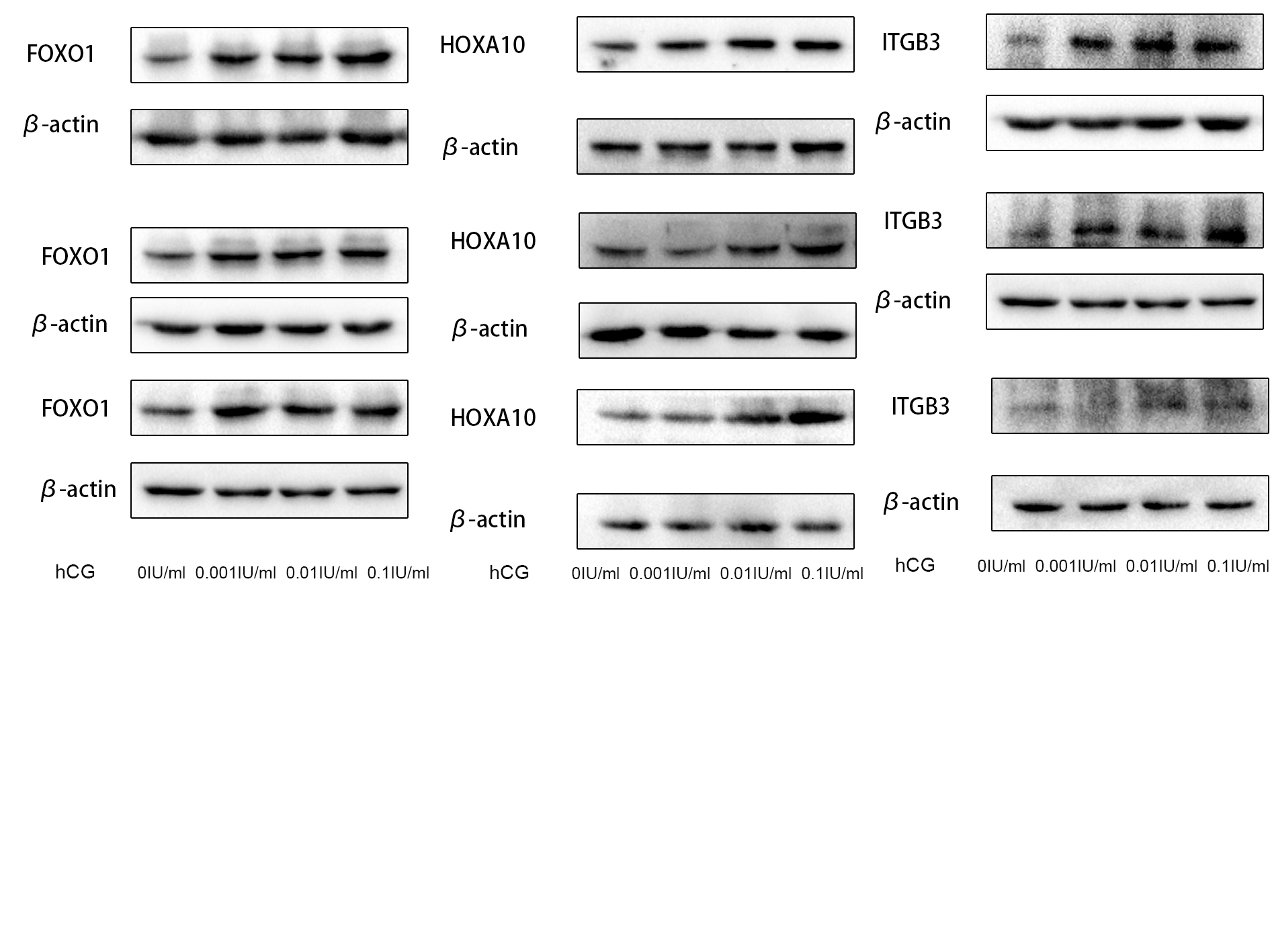

Supplement: Supplementary file 1 — Supplementary Material 1. [file 12958_2024_1205_MOESM1_ESM.zip › WESTERN/FOXO1,ITGB3 and HOXA10-ESCs.tif]

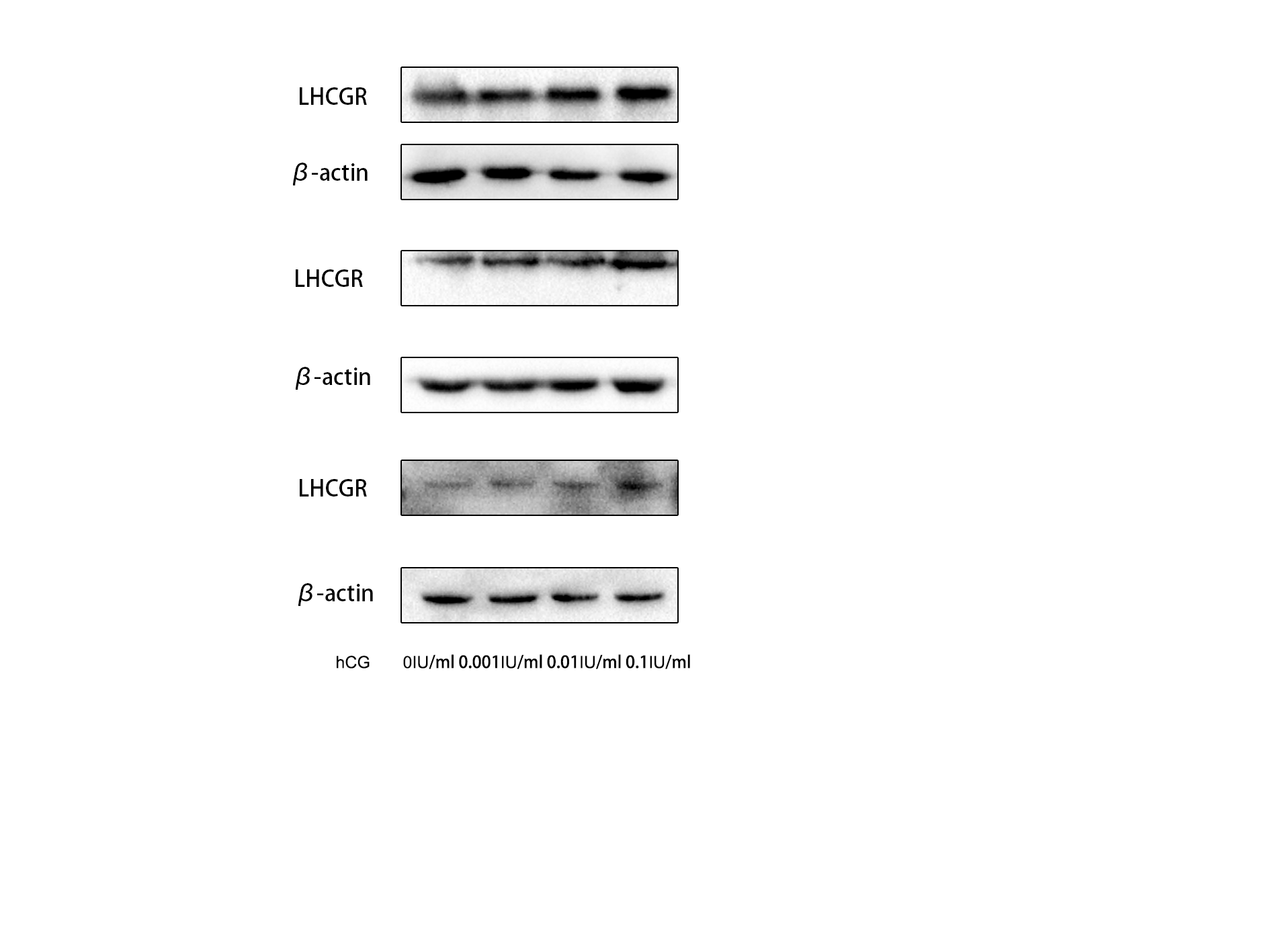

Supplement: Supplementary file 1 — Supplementary Material 1. [file 12958_2024_1205_MOESM1_ESM.zip › WESTERN/LHCGR-ESCs.tif]

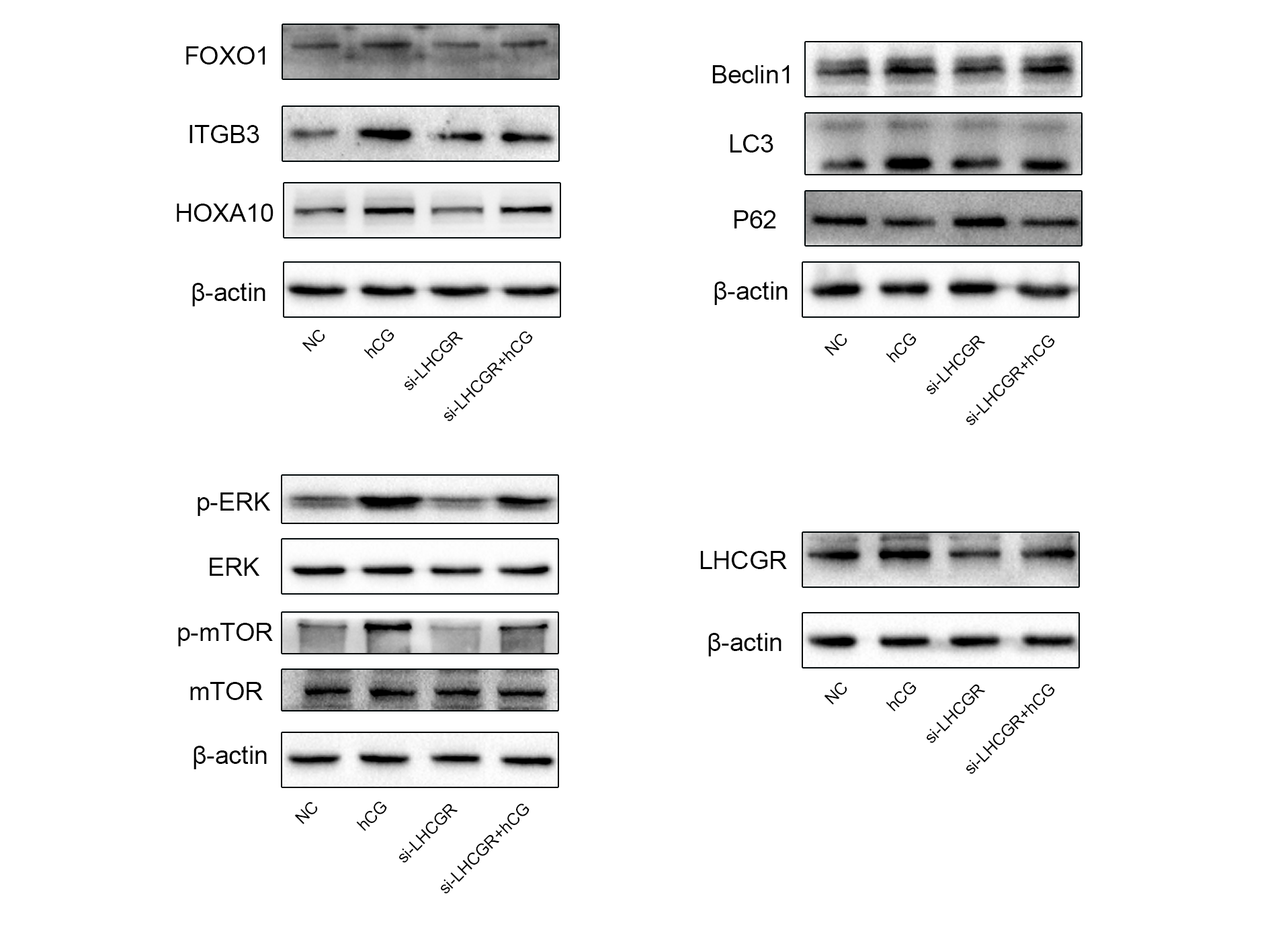

Supplement: Supplementary file 1 — Supplementary Material 1. [file 12958_2024_1205_MOESM1_ESM.zip › WESTERN/siRNA-1.tif]

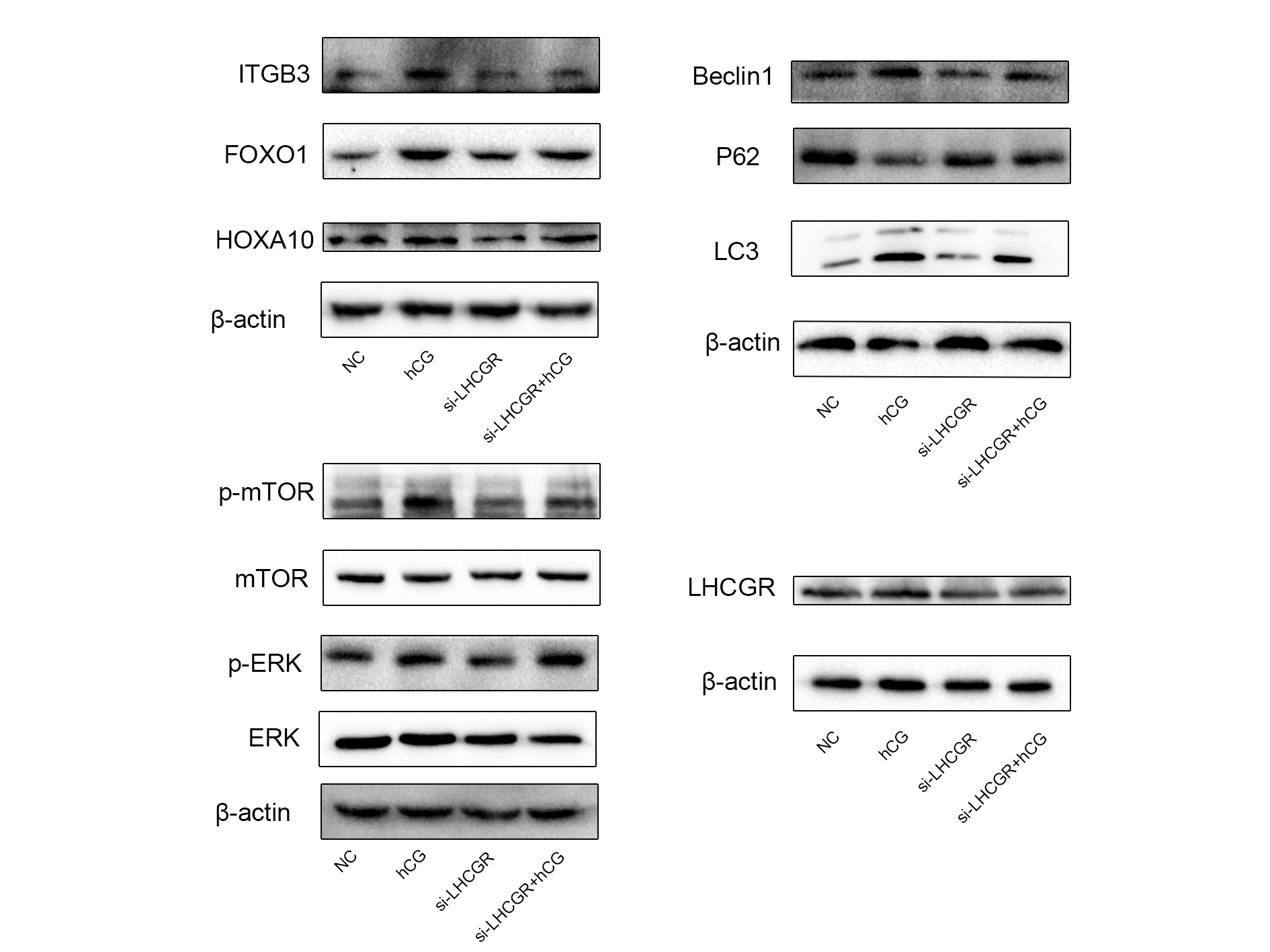

Supplement: Supplementary file 1 — Supplementary Material 1. [file 12958_2024_1205_MOESM1_ESM.zip › WESTERN/siRNA-2.tif]

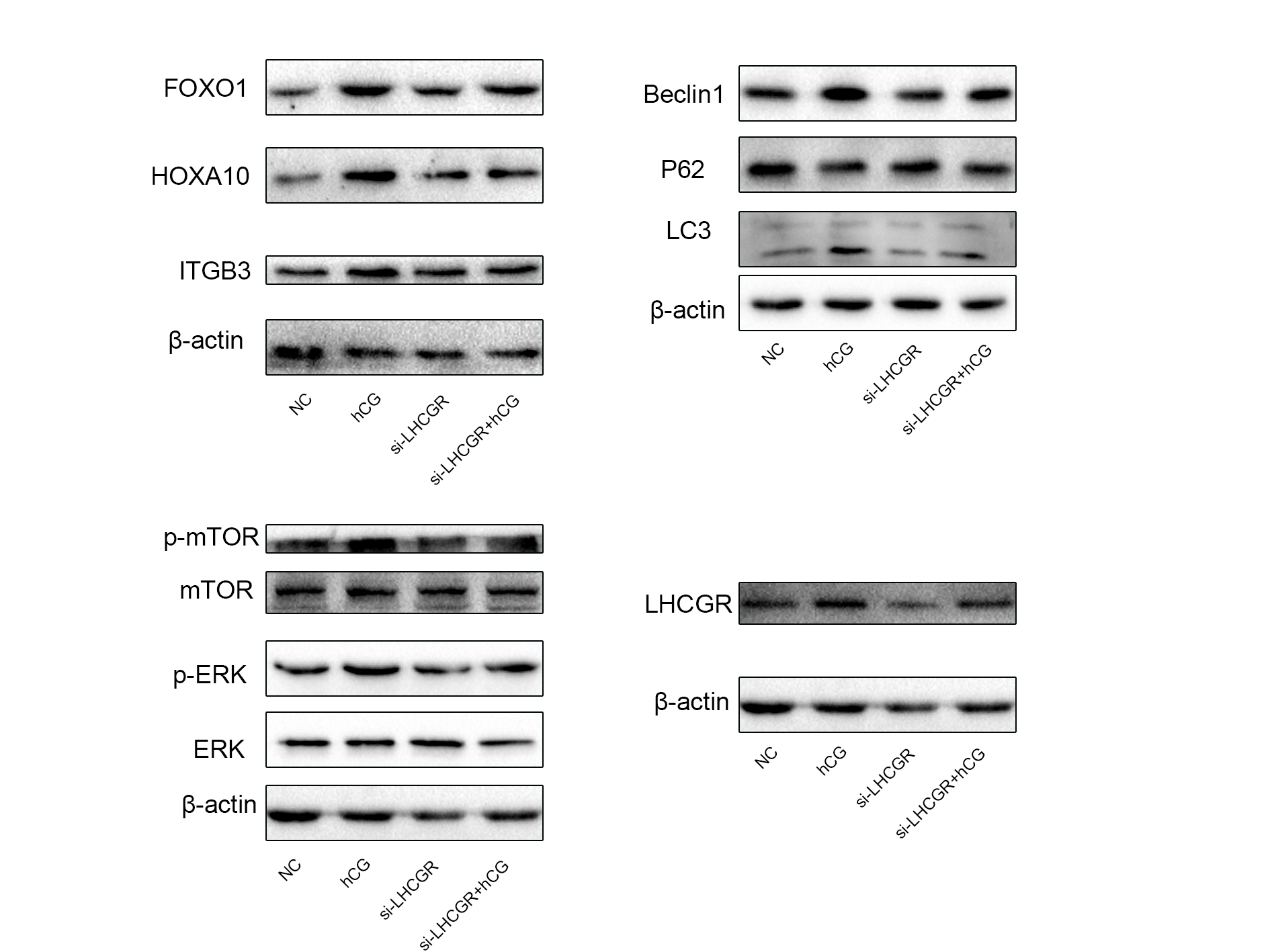

Supplement: Supplementary file 1 — Supplementary Material 1. [file 12958_2024_1205_MOESM1_ESM.zip › WESTERN/siRNA-3.tif]
